# The effect of lesion filling on brain network analysis in multiple sclerosis using structural magnetic resonance imaging

**DOI:** 10.1186/s13244-022-01198-4

**Published:** 2022-03-28

**Authors:** Chris W. J. van der Weijden, Milena S. Pitombeira, Yudith R. A. Haveman, Carlos A. Sanchez-Catasus, Kenia R. Campanholo, Guilherme D. Kolinger, Carolina M. Rimkus, Carlos A. Buchpiguel, Rudi A. J. O. Dierckx, Remco J. Renken, Jan F. Meilof, Erik F. J. de Vries, Daniele de Paula Faria

**Affiliations:** 1 Department of Nuclear Medicine and Molecular Imaging, University of Groningen, University Medical Center Groningen, Hanzeplein 1, 9713GZ Groningen, The Netherlands; 2 Department of Neurology, Faculdade de Medicina da Universidade de Sao Paulo, Sao Paulo, Brazil; 3 Department of Neurology, Clínica Universidad de Navarra, 31008 Pamplona, Spain; 4 Department of Radiology and Oncology, Faculdade de Medicina da Universidade de Sao Paulo, Sao Paulo, Brazil; 5 Department of Neuroscience, University Medical Center Groningen, University of Groningen, Hanzeplein 1, Groningen, The Netherlands; 6 Department of Biomedical Sciences of Cells and Systems, University of Groningen, University Medical Center Groningen, Hanzeplein 1, Groningen, The Netherlands; 7 Department of Neurology, Martini Ziekenhuis, Groningen, The Netherlands

**Keywords:** Graph theoretical network analysis, Multiple sclerosis, Lesion filling, Demyelinating diseases, Neurodegenerative diseases

## Abstract

**Background:**

Graph theoretical network analysis with structural magnetic resonance imaging (MRI) of multiple sclerosis (MS) patients can be used to assess subtle changes in brain networks. However, the presence of multiple focal brain lesions might impair the accuracy of automatic tissue segmentation methods, and hamper the performance of graph theoretical network analysis. Applying “lesion filling” by substituting the voxel intensities of a lesion with the voxel intensities of nearby voxels, thus creating an image devoid of lesions, might improve segmentation and graph theoretical network analysis. This study aims to determine if brain networks are different between MS subtypes and healthy controls (HC) and if the assessment of these differences is affected by lesion filling.

**Methods:**

The study included 49 MS patients and 19 HC that underwent a T1w, and T2w-FLAIR MRI scan. Graph theoretical network analysis was performed from grey matter fractions extracted from the original T1w-images and T1w-images after lesion filling.

**Results:**

Artefacts in lesion-filled T1w images correlated positively with total lesion volume (*r* = 0.84, *p* < 0.001) and had a major impact on grey matter segmentation accuracy. Differences in sensitivity for network alterations were observed between original T1w data and after application of lesion filling: graph theoretical network analysis obtained from lesion-filled T1w images produced more differences in network organization in MS patients.

**Conclusion:**

Lesion filling might reduce variability across subjects resulting in an increased detection rate of network alterations in MS, but also induces significant artefacts, and therefore should be applied cautiously especially in individuals with higher lesions loads.

**Supplementary Information:**

The online version contains supplementary material available at 10.1186/s13244-022-01198-4.

## Key points

- Lesion filling increases the sensitivity of T1w-derived network analysis in multiple sclerosis (MS).
- Lesion filling produces more artefacts in patients with greater lesion volumes.
- Network parameters are more affected in relapse-remitting than progressive MS.

## Background

Multiple sclerosis (MS) is an inflammatory demyelinating disease, and the most common neurodegenerative disease among young adults [1]. The disease is characterized by apparently randomly located inflammatory lesions within the central nervous system (CNS), resulting in a variety of clinical manifestations among patients [1]. The relapsing–remitting multiple sclerosis phenotype (RRMS) presents acute clinical manifestations, the so-called relapses, followed by a period of full or partial recovery and stable disability between episodes, whereas the progressive phenotype (PMS) is defined when a consistent increasing in neurological disability is confirmed independent of relapses [2–5]. Despite the high heterogeneity within MS pathology due to the random location of lesions, there are also common symptoms. These symptoms may include a decreased visual function, bladder dysfunction, and impaired motor and sensory functions [6]. Although allocation of symptoms to specific spinal cord dysfunction is generally supported by a myelopathy visible on MRI, studies have not yet been able to consistently link symptoms to individual cerebral lesions visible on MRI. A general explanation for this discrepancy is the existence of functional cerebral networks, which implies that lesions in different parts of a network can result in similar symptoms. A progressive disruption of functional networks during disease progression could explain the increase in disability in later stages of MS.

To investigate this hypothesis, graph theoretical network analysis could be used for different phenotypes or stages of MS. According to graph theory, the connections between brain regions can be presented in a graph, in which the brain regions are represented as nodes and the interactions as lines (edges). These interactions can be used to calculate various parameters, like path length, which describes the number of edges between two brain regions. These parameters are calculated per subject and can then be compared between two groups. In principle, any imaging modality could be used for graph theory, but the clinical meaning and relevance is highly depending on the imaging used as input. So far, mainly diffusion tensor imaging (DTI) has been used for structural network analysis in MS [7]. DTI assesses white matter (WM) tractography and therefore could be used to determine white matter connectivity. Grey matter (GM) connectivity in MS could be studied using parameters obtained from T1-weighted (T1w) MRI, such as cortical volume, cortical thickness, or grey matter fraction [7]. Studies showed high similarities between DTI and T1w results obtained with graph theoretical network analysis in MS patients [8–10]. A challenge for network analysis using either DTI or T1w MRI in MS is the random location of lesions, and for T1w especially the effect of juxtacortical lesions on GM segmentation. A method to cope with the variation in lesion load and distribution is the application of lesion filling [11]. Lesion filling replaces the voxel intensities in lesions with the voxel intensity of surrounding tissue, resulting in an image without apparent lesions, and thus devoid of the pathological signal intensity variations. Some studies performing network analysis in MS apply lesion filling [12, 13], whereas others do not [8, 14], and therefore, there is no clear consensus regarding the application of lesion filling.

Studies on structural networks using graph theoretical network analysis in MS show inconsistent findings. Some studies, assessing either GM (T1w) or WM (DTI) connectivity, found a decrease in global and local efficiency of the network in MS patients [8–10, 15], whereas other studies found an increase [12, 14, 16]. This discrepancy between studies might reflect the deteriorating reorganizational properties of the brain to minimise clinical disability during disease progression. These controversies regarding WM and GM connectivity could therefore be due to differences in study populations, especially with regard to disease duration and disease subtype. Dedicated studies on the effects of different MS phenotypes or disease stages on connectivity measures could help to resolve this issue.

Our study aims to (1) determine the effect of lesion filling on graph theoretical network parameters derived from T1w MRI, (2) identify which regions are important for MS pathogenesis by assessing abnormalities in GM connectivity, and (3) investigate if GM connectivity differs between RRMS and PMS patients.

## Methods

### Participants

Seventy-five subjects were recruited at the Medical Faculty of the University of São Paulo, which consisted of 24 healthy controls (HC), 30 RRMS patients, and 21 PMS patients. Inclusion criteria for the healthy controls were age between 18 and 65 years and at least 4 years of education. Patients were diagnosed with clinically defined MS according to the revised McDonald criteria [17], were relapse free and had not received steroid treatment for at least 30 days before MRI scanning. Exclusion criteria were any major medical conditions that prevented MRI acquisition (i.e. pregnancy, renal, cardiac, or hepatic insufficiency) or presence of severe psychiatric disorders. Hence, 3 healthy subjects were excluded due to unexpected comorbidities, 1 participant withdrew from the study and did not allow further use of data, 1 participant was scanned with a different head coil due to dysphagia, 1 subject did not conclude the whole protocol, and 1 participant received intravenous steroid treatment 5 days before MRI acquisition which was reported only after the scan. All participants signed the informed consent form to participate in the study. The study was conducted according to the Declaration of Helsinki and subsequent revisions, and was approved by the medical ethics committee of the University of São Paulo (protocol 3.256.558). Differences in demographics were assessed with nonparametric tests where applicable.

### Image acquisition

MRI scans of the brain were performed on a 3 T SIGNA PET-MRI scanner (General Electric Company) with a 24-channel head coil. The protocol comprised of a 3D-T1w (TR/TE/TI = 7.664/3.112/600 ms, voxel size 1 × 0.5 × 0.5 mm) and a 3D-T2w FLAIR (TR/TE/TI = 6500/141.213/1905 ms, voxel size 1.3 × 0.5 × 0.5 mm) sequence.

### Image processing

T2w-FLAIR images were co-registered to the 3D-T1w images. The lesion growth algorithm (LGA) of the lesion segmentation toolbox (LST) in SPM12 (Wellcome Trust Centre for Neuroimaging, Institute of Neurology, London, 2014) was used with a kappa of 0.3 to perform lesion segmentation on T1w and T2w-FLAIR images, resulting in a lesion probability map. Subsequently, these lesion probability maps were used for lesion filling of T1w images by using local information (i.e. filling the lesion with the intensity of adjacent voxels), allowing accurate lesion filling even in images that are corrupted by bias field [18]. Original and lesion-filled T1w MRI images were processed by GM, WM and cerebrospinal fluid (CSF) segmentation and spatial normalization to the Montreal Neurological Institute (MNI) space using the tissue probability maps in SPM12 [19]. The accuracy of the co-registration, segmentation, lesion filling, and normalisation was checked by visual inspection. Network analysis was performed both on the original T1w data and on the T1w data with lesion filling.

### Network-analysis

**Fig. 1:**
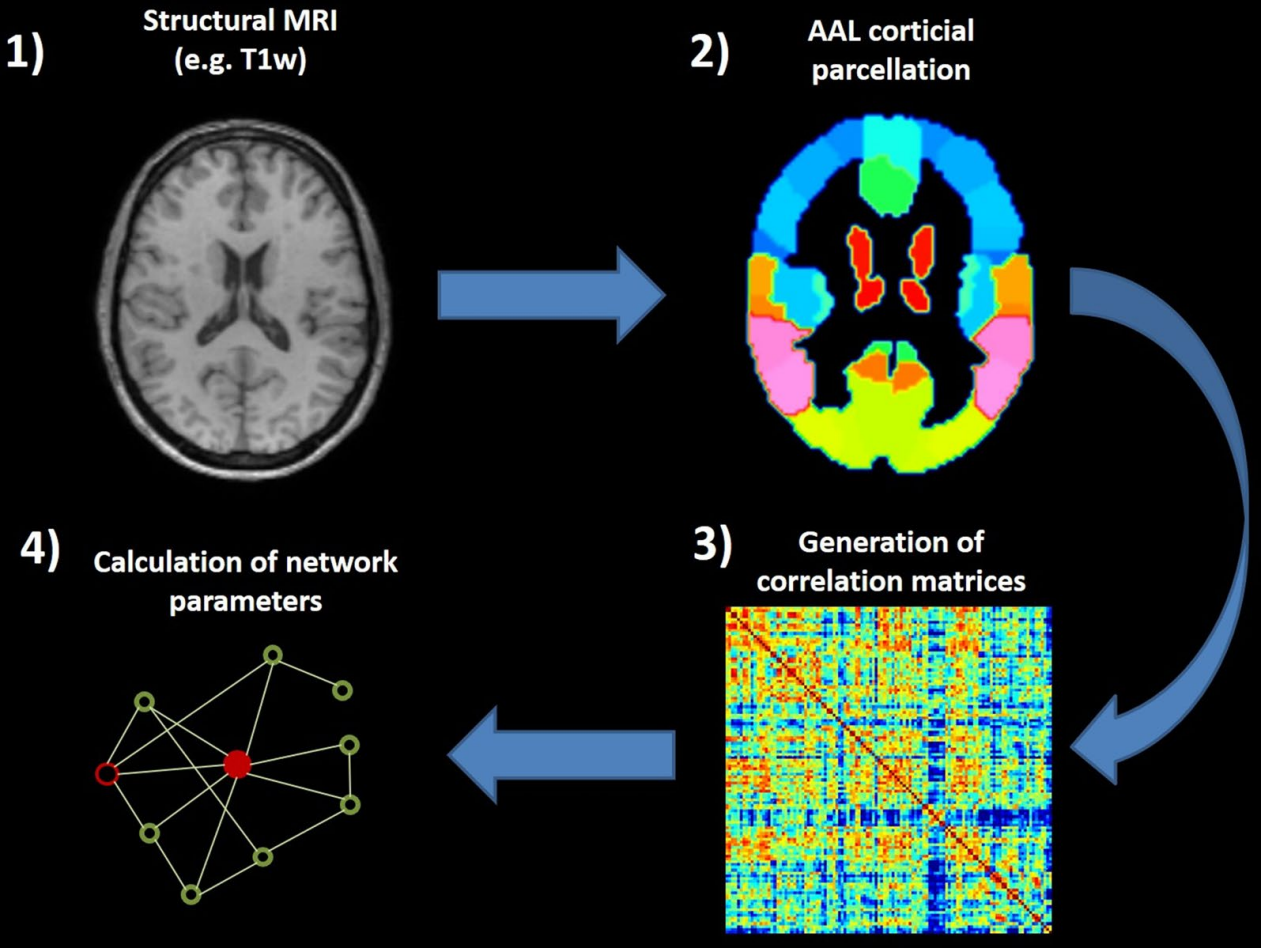
Flowchart of the graph theoretical network analysis. AAL: automated anatomical labelling

Graph theoretical network analysis comprises 4 main steps (Fig. 1): (1) defining the appropriate nodes, (2) estimation of a continuous measure of association between nodes, (3) generation of an association matrix, and (4) calculating the network parameters of interest. Using the 116 regions of interest (ROIs) within the automated anatomical labelling (AAL) atlas within the Wake Forest University (WFU) Pickatlas tool in SPM12 [20], grey matter fractions were extracted from the GM segmentation derived of ROIs in the T1w images. The grey matter fractions corresponding to the 116 AAL ROIs were used as nodes. Association matrices were generated with Brain Analysis using Graph theory (BRAPH) [21] from Spearman correlations to assess the inter-regional associations (the edges between nodes): a higher correlation indicated a stronger inter-region association. Spearman correlations were used due to their insensitivity to outliers and skewed distributions. Negative correlations were set to zero, because not all network parameters can be calculated in the presence of negative correlation coefficients. In recent years, there have been advances in using weighted graphs and increasing calls for their use across all neuroimaging modalities [22]. Therefore, several network parameters (Table 1) were calculated per group using weighted undirected graphs. Global parameters are calculated over the whole brain and therefore provide information regarding the integrity of the whole brain network. However, nodal parameters are calculated for individual brain regions, and thus provide information regarding the network integrity of the individual brain regions.

**Table 1:** Studied network parameters and their definitions

Measure	Definition	Nodal level	Global level
Degree	Number of connections to a node	x	x*
Strength	Sum of the weight of all connections to a node	x	x*
Path length	Lowest number of connections between two nodes	x	x*
Clustering coefficient	The fraction of a node’s neighbours that are also neighbour between each other	x	x*
Global efficiency	Average inverse of shortest path length	x	x*
Local efficiency	Global efficiency of a node regarding its neighbourhood	x	x*
Within module degree z-score	Within module degree of centrality	x	
Participation	The diversity of intermodular interconnections of individual nodes	x	
Transitivity	The probability of interconnectivity of adjacent nodes		x
Modularity	Degree to which the graph can be subdivided into multiple small-world networks		x
Assortativity coefficient	Correlation coefficient between degrees/strengths of all nodes on two opposite ends of a connection		x
Small-worldness	The ratio of clustering coefficient on global level and the clustering coefficient of a random graph divided by the ratio of the average path length on global level and the average path length of a random graph		x

*Network parameters at global level were calculated by averaging the outcome of nodal measures over all ROI’s

Nonparametric permutation tests were applied to both data derived from original T1w MRI and data derived from lesion filled T1w MRI to assess the significance of the differences in the network parameters between two groups (HC vs. MS total, HC vs. RRMS, HC vs. PMS, and RRMS vs. PMS) [23]. The tests were performed by first determining the differences between two groups for each network parameter. Then, the subjects were permutated between the two groups, and the network parameters (e.g. strength) were calculated again for the permuted groups (containing a mix of subjects of the two groups). This process was repeated 1000 times. After each permutation, the differences in network parameters between the permutated groups were determined, resulting in distributions of between-group differences and 95% confidence intervals (CIs) of the differences per network parameter. HC (or RRMS in case of RRMS vs. PMS) were used as a reference for the 95% CIs. If the distribution of a particular MS group was outside the 95% CI of the reference group (HC or RRMS), the difference was considered significant at a global level. The results on a nodal level were corrected for multiple comparisons using the false discovery rate (FDR) Benjamini–Hochberg procedure [24] with a *q* of 0.05 to correct for the number of regions that were tested. Differences were considered to be truly significant and not dependent on chance, if the uncorrected *p* values were both ≤ 0.05 and equal to or smaller than the FDR corrected *p* values.

## Results

### Study population

Only patients off-steroid treatment without comorbidities, who correctly concluded the whole MRI protocol, were included in the graph theoretical network analysis. This led to a final inclusion of 19 HC, 30 RRMS (disease duration 9.3y ± 5.9), and 19 PMS patients (disease duration 11.8y ± 6.8), of which 11 had primary and 8 secondary PMS (Table 2). Patients with RRMS were significantly younger (age 35.7 vs. 49.3 years, *t* = − 5.7, *p* < 0.001) and had significantly lower disability scores than PMS patients (EDSS 2.7 vs 6.3, *U* = 556, *p* < 0.001), but had similar numbers and volumes of white matter lesions, years of education, and disease duration. Surprisingly, a small number of non-specific brain lesions that do not correspond with a specific diagnosis or aetiology were detected in HC.

**Table 2:** Study population characteristics. Age, education, disease duration, EDSS, amount of lesions, lesion volume are presented as mean (± SD)

	HC	MS total	RRMS	PMS
Number of participants	19	49	30	19
Gender (%male)	21.1	34.7	29.0	42.1
Age (years)	41.3 (± 12.8)	41.0 (± 10.5)	35.7 (± 7.6)*	49.3 (± 8.9)*
Education (years)	13.9 (± 3.8)	13.1 (± 3.9)	13.7 (± 3.5)	12.3 (± 4.4)
Disease duration (years)	0	10.3 (± 6.3)	9.3 (± 5.9)	11.8 (± 6.8)
EDSS	0 (± 0)	4.1 (± 2.1)	2.7 (± 1.4)*	6.3 (± 0.8)*
Number of lesions	1.9 (± 2.7)	15.7 (± 8.5)	16.3 (± 9.3)	14.7 (± 7.3)
Total lesion volume (ml)	0.3 (± 0.8)	23.9 (± 8.5)	15.3 (± 18.7)	25.5 (± 29.9)

Significant differences between RRMS and PMS patients are indicated with an asterisk. *p < 0.001

### Global network topology based on original T1w MRI data

**Fig. 2:**
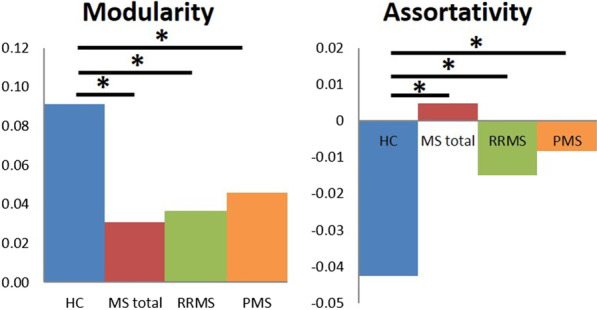
Group differences of T1w MRI data without lesion filling in the global graph theoretical network parameters modularity and assortativity. Comparisons were made for HC versus MS total, HC versus RRMS, HC versus PMS, and RRMS versus PMS. Significant differences are indicated with an asterisk *

Modularity and assortativity were the only global network parameters derived from T1w images without lesion filling that showed significant differences between groups. Modularity was significantly higher in HC than in MS patients. The T1w-derived assortativity (correlation coefficient, indicating whether similar nodes are connected to each other) was significantly lower in HC than in MS patients (Table 3, Fig. 2). Both parameters did not significantly differ between RRMS and PMS patients.

**Table 3:** Differences in global graph theoretical network parameters between groups. The corresponding 95% confidence intervals (CI) are presented between brackets

Network parameter	MRI	HC versus MS total	HC versus RRMS	HC versus PMS	RRMS versus PMS
Average degree	Original T1w	3.17 (− 1.35–7.37)	3.93 (− 2.68–7.60)	0.29 (− 4.50–4.61)	− 3.64 (− 6.50–3.59)
	Lesion-filled T1w	**5.16** **(**− **0.01**–2.31)*	**5.17** **(**− **0.38**–2.68)*	**4.48** **(**− **1.93**–1.80)*	− 0.69 (− 2.07–0.29)
Average strength	Original T1w	17.0 (− 17.2–21.4)	16.0 (− 21.0–23.1)	14.5 (− 21.5–21.8)	− 1.5 (− 23.0–19.9)
	Lesion-filled T1w	**27.9** **(**− **15.0**–20.0)*	**30.1** **(**− **20.4**–23.4)*	**19.0** **(**− **18.1**–18.5)*	− 11.1 (− 20.3–17.7)
Average path length	Original T1w	− 0.46 (− 0.66–0.53)	− 0.46 (− 0.75–0.67)	− 0.40 (− 0.64–0.60)	0.06 (− 0.63–0.64)
	Lesion-filled T1w	− **0.86** **(**− **0.57**–0.34)*	− **0.92** **(**− **0.63**–0.54)*	− **0.60** **(**− **0.49**–0.46)*	0.32 (− 0.48–0.55)
Global efficiency	Original T1w	0.11 (− 0.14–0.14)	0.10 (− 0.16–0.15)	0.10 (− 0.16–0.16)	0.00 (− 0.15–0.17)
	Lesion-filled T1w	**0.20** **(**− **0.12**–0.15)*	**0.22** **(**− **0.17**–0.18)*	0.13 (− 0.14–0.14)	− 0.08 (− 0.15–0.14)
Local efficiency	Original T1w	1.26 (− 1.91–1.78)	1.16 (− 1.91–1.72)	1.22 (− 1.93–2.00)	0.06 (− 1.66–2.01)
	Lesion-filled T1w	**2.33** **(**− **1.68**–2.01)*	**2.60** **(**− **2.29**–2.26)*	1.50 (− 1.91–2.14)	− 1.10 (− 2.15–2.00)
Clustering	Original T1w	0.17 (− 0.14–0.19)	0.15 (− 0.18–0.20)	0.14 (− 0.20–0.19)	− 0.01 (− 0.19–0.17)
	Lesion-filled T1w	**0.26** **(**− **0.12**–0.19)*	**0.28** **(**− **0.19**–0.21)*	0.17 (− 0.17–0.17)	− 0.11 (− 0.20–0.15)
Transitivity	Original T1w	0.25 (− 0.22–0.30)	0.22 (− 0.27–0.29)	0.22 (− 0.28–0.30)	0.00 (− 0.31–0.26)
	Lesion-filled T1w	**0.39** **(**− **0.19**–0.27)*	**0.42** **(**− **0.26**–0.34)*	0.26 (− 0.24–0.26)	− 0.16 (− 0.30–0.22)
Modularity	Original T1w	− **0.061** **(**− **0.060**–0.014)*	− **0.055** **(**− **0.054**–0.030)*	− **0.045** **(**− **0.040**–0.038)*	0.010 (− 0.029–0.054)
	Lesion-filled T1w	− **0.071** **(**− **0.044**–0.011)*	− **0.069** **(**− **0.050**–0.028)*	− **0.054** **(**− **0.026**–0.027)*	0.015 (− 0.017–0.039)
Assortativity	Original T1w	**0.047** **(**− **0.024**–0.031)*	**0.028** **(**− **0.022**–0.025)*	**0.034** **(**− **0.027**–0.026)*	0.007 (− 0.051–0.048)
	Lesion-filled T1w	**0.024** **(**− **0.001**–0.023)*	**0.025** **(**− **0.008**–0.024)*	0.014 (− 0.019–0.019)	− 0.012 (− 0.018–0.008)
Small-worldness	Original T1w	0.010 (− 0.027–0.064)	0.019 (− 0.038–0.064)	− 0.011 (− 0.048–0.051)	− 0.030 (− 0.060–0.053)
	Lesion-filled T1w	**0.065 (**− 0.011–0.056)*	**0.069 (**− 0.028–0.054)*	0.034 (− 0.039–0.041)	− 0.035 (− 0.053–0.027)

Differences relative to the healthy controls are statistically significant when the differences fall outside the 95% CI. Significant differences are indicated with an asterisk *. No statistically significant differences were observed between the RRMS and PMS group

### Lesion filling

**Fig. 3:**
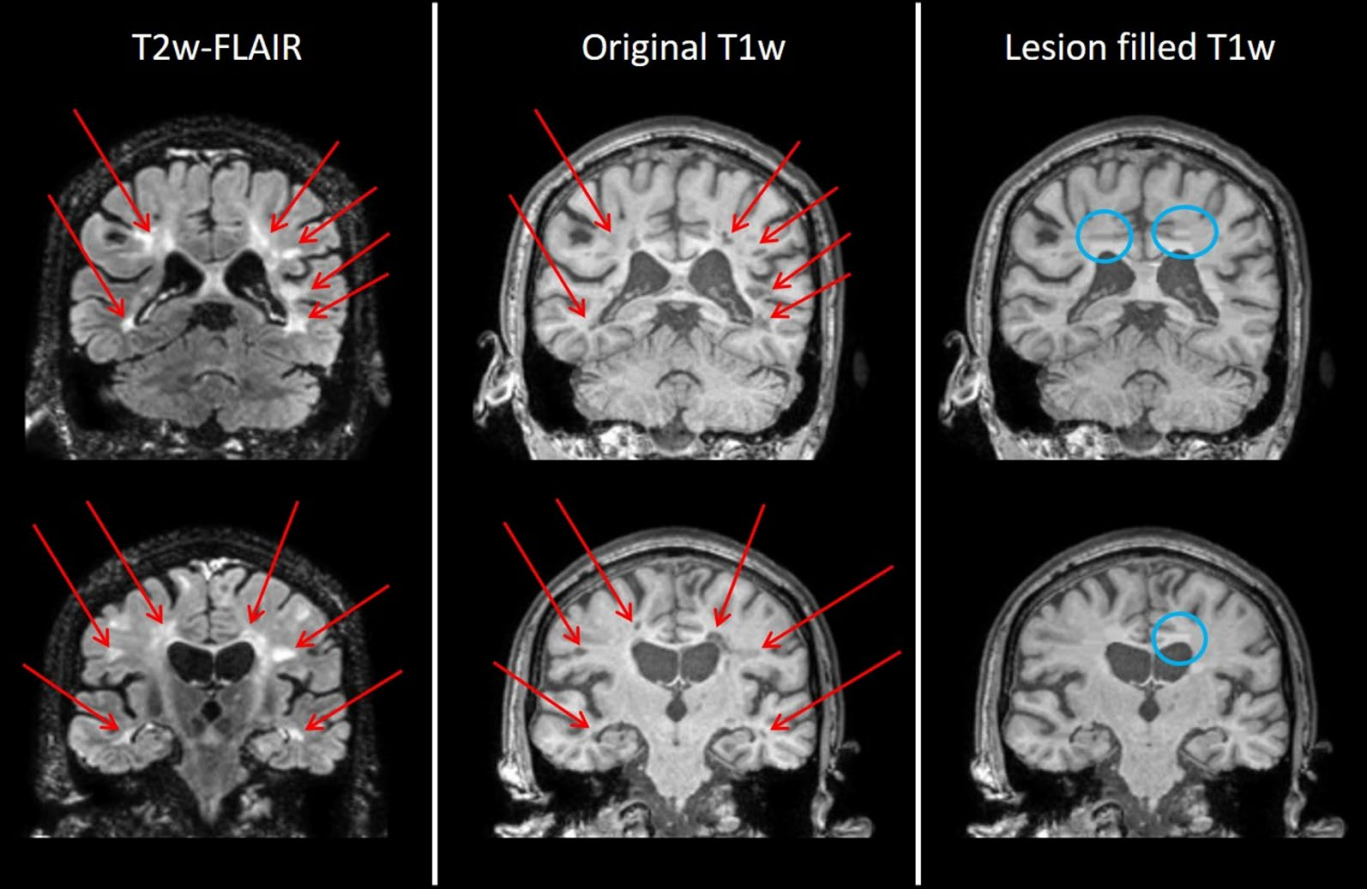
The effects of lesion filling on T1w MRI illustrating artefacts due to lesion filling. Red arrows depict the locations of the lesions; blue circles depict lesion filling artefacts. The upper row shows some substantial artefacts due to lesion filling, composed of grey matter tissue allocation in the middle of white matter regions, the lower row shows a minor artefact, having blunt white matter edges

**Fig. 4:**
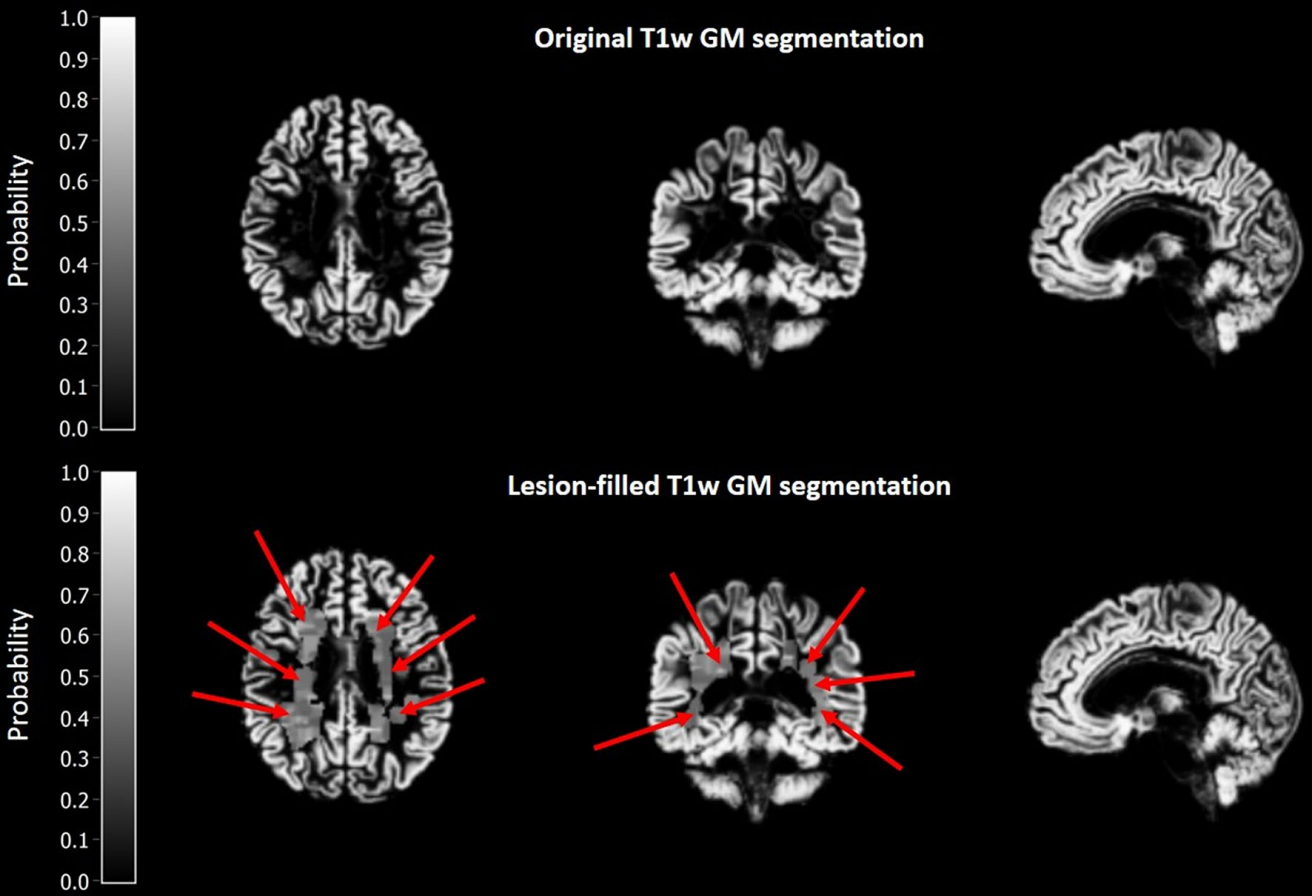
GM segmentation of both original T1w and lesion-filled T1w MRI

Lesion filling on T1w MRI data was applied to evaluate the effect of lesion filling on the graph theoretical network outcome parameters. In total, 804 lesions were detected across the study population. Lesion-filling was accompanied with a total amount of 233 artefacts, of which 79 were substantial (wrong classification of tissue) and 152 minor (Fig. 3). Most artefacts generated by lesion filling were observed for lesions at the interface between tissues (e.g. interface between WM and CSF, or WM and GM) and lesions with a large volume. Quantitative assessment using Spearman correlation, revealed a correlation between total lesion volume and the number of lesion filling artefacts (*r* = 0.84, *p* < 0.001). An example of the substantial effects of the lesion filling artefacts on GM segmentations is displayed in Fig. 4. All images were included in the network analysis, irrespective of the extent of lesion filling artefacts.

### Global network topology of lesion filled MRI

**Fig. 5:**
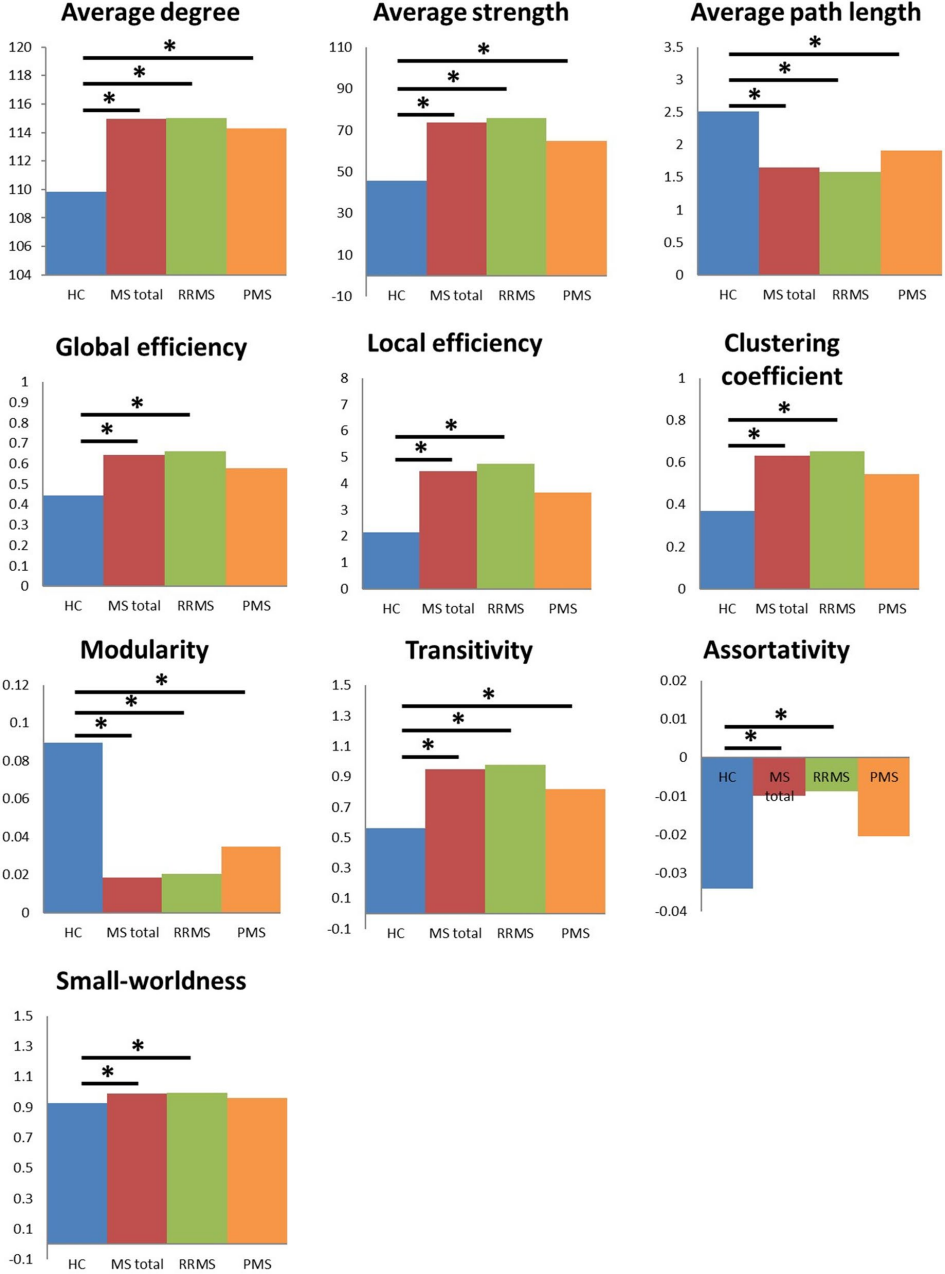
Group differences using lesion filled T1w data in global graph theoretical network parameters of structural connectomes. Comparisons that have been made were HC versus MS total, HC versus RRMS, HC versus PMS, and RRMS versus PMS. Significant differences are indicated with an asterisk *

Network analysis of lesion-filled T1w images (Fig. 5 and Table 3) showed significant differences in all global network parameters between the HC and total MS group. In particular, the average degree, average strength, global and local efficiency, clustering coefficient, transitivity, assortativity, and small-worldness were significantly increased in the total MS group, whereas the average path-length and modularity were significantly decreased (Table 3). Comparison of HC with RRMS showed similar significant differences for all parameters. The PMS group only significantly differed from HC for the parameters average degree, average strength, transitivity, path-length and modularity. For all network parameters, the scores of the PMS group deviated less from HC than those of the RRMS group. No statistically significant differences between the RRMS and PMS group were found for any of the parameters.

### Nodal network topology with and without lesion filling

Regional values for nodal network topology are presented in Additional file 1: Table S1–S6. For a better overview, FDR corrected significant nodes (*q* = 0.05) were compiled together based on their anatomical location (Table 4). When assessing network parameters derived from original MRI data at the level of individual nodes, the degree was statistically significant decreased in frontal lobe of PMS compared to HC, and increased in posterior fossa of PMS compared to HC (Additional file 1: Table S1). No significant differences between groups were observed for any other parameter after false-discovery rate (FDR) correction. In contrast, network analysis of lesion-filled T1w images showed statistically significant differences between groups for many regional network topologies, even after FDR correction (Table 4 and Additional file 1: Table S2–S6).

**Table 4:** Compilation of regional differences in network topology derived from lesion-filled T1w MRI images. The results for the comparisons of the different MS groups with healthy controls are provided. The comparison RRMS versus PMS did not yield any significant results

MS group	Frontal lobe	Temporal lobe	Parietal lobe	Occipital lobe	Central structures	Cingulate gyri	Posterior fossa
Path length							
MS total	↓	↓	↓	↓	n.s	↓	↓
RRMS	↓	↓	↓	↓	n.s	↓	↓
PMS	n.s	↓	↓	↓	n.s	n.s	↓
Degree							
MS total	↑	↑	↑	↑	↑	↑	↑
RRMS	↑	↑	↑	↑	↑	↑	↑
PMS	↑	↑	↑	↑	↑	↑	↑
Strength							
MS total	↑	↑	↑	↑	n.s	↑	↑
RRMS	↑	↑	n.s	n.s	n.s	n.s	↑
PMS	n.s	n.s	n.s	n.s	n.s	n.s	n.s
Global efficiency							
MS total	n.s	n.s	n.s	n.s	n.s	n.s	↑
RRMS	n.s	n.s	n.s	n.s	n.s	n.s	↑
PMS	n.s	n.s	n.s	n.s	n.s	n.s	n.s
Local efficiency							
MS total	n.s	n.s	n.s	n.s	n.s	n.s	n.s
RRMS	n.s	n.s	n.s	n.s	n.s	n.s	n.s
PMS	n.s	n.s	n.s	n.s	n.s	n.s	n.s
Clustering							
MS total	↑	↑	↑	↑	n.s	↑	↑
RRMS	n.s	n.s	n.s	n.s	n.s	n.s	↑
PMS	n.s	n.s	n.s	n.s	n.s	n.s	n.s
Within module degree z-score							
MS total	n.s	n.s	n.s	n.s	n.s	n.s	n.s
RRMS	n.s	n.s	n.s	n.s	n.s	n.s	n.s
PMS	n.s	n.s	n.s	n.s	n.s	n.s	n.s
Participation							
MS total	n.s	n.s	n.s	n.s	n.s	n.s	n.s
RRMS	n.s	n.s	n.s	n.s	n.s	n.s	n.s
PMS	n.s	n.s	n.s	n.s	n.s	n.s	n.s

*n.s. = not significant, ↑ is significantly increased compared to HC, ↓ is significantly decreased compared to HC

The nodal degree was increased in all brain areas in all MS groups, when compared to HC. Nodal strength, global efficiency, and cluster coefficient in posterior fossa were higher in the total MS and RRMS groups than in HC, but was not affected in PMS. In all MS groups, path length in the temporal lobe, parietal lobe, occipital lobe, and posterior fossa was lower than in HC. In the total MS and RRMS groups, path length was also decreased in the frontal lobe and in the insula and cingulate gyri.

Assessing the regions more in detail (Additional file 1: Table S2–S6), most network parameters were affected in the right Heschl gyrus, cerebellum crus 2, and right cerebellum part 7b in all MS groups, whereas nodal degree, nodal strength, path length, and clustering coefficient were also affected in right superior occipital gyrus, and left and right middle temporal gyrus.

Nodal degree in the left supplementary motor area was only significantly decreased in PMS patients (108) compared to HC (112), but not in RRMS patients (115). Furthermore, both the nodal degree and path length in the left calcarine fissure (CAL.L), left dorsolateral cingulate gyrus (DCG.L), and right paracentral lobule (PCL.R) were only significantly affected in RRMS (CAL.L: 115; 1.36, DCG.L: 115; 1.45, PCL.R: 115; 1.55, respectively) but not in PMS patients (CAL.L: 115; 1.69, DCG.L: 113; 2.10, PCL.R: 110; 2.40, respectively) compared to HC (CAL.L: 112; 2.53, DCG.L: 110; 2.57, PCL.R: 105; 2.96, respectively). The nodal degree of the right putamen was only affected in RRMS patients (115) compared to HC (97), and not in PMS patients (100).

## Discussion

MS is a very heterogeneous disease with pathogenesis and symptomatology varying per individual. However, similarities among symptoms and disability are found, suggesting the presence of a common pathway in the pathogenesis. Therefore, this study aimed to find regions that are affected in MS, which might indicate the region’s importance in MS pathogenesis. In addition, we investigated whether regions are differentially affected between MS phenotypes and, most importantly, what the effect of lesion filling is on the calculation of graph theoretical network parameters derived from structural T1w MRI images. Although lesion-filling introduced substantial artefacts for some lesions, it has significantly reduced the heterogeneity between MS patients, enabling detection of more differences in network parameters in a relatively small dataset. In general, the difference in global network parameters between the RRMS and HC group was larger than between the PMS and HC group, but no significant differences between the RRMS and PMS group were observed. Both in PMS and RRMS, all investigated network parameters were affected on nodular level in the right Heschl gyrus, cerebellum crus 2, and cerebellum part 7b. This indicates the importance of these regions in MS pathogenesis.

Graph theoretical network analysis on original T1w MRI data detected only a few differences between groups on a global level. The only global effects observed were a reduced modularity and a higher assortativity in MS patients, when compared to HC. Nodes with a high degree tend to be connected to nodes with a low degree, which results in a low assortativity [14, 25]. The increased assortativity in MS patients therefore suggests a more random network, which is supported by the decreased modularity. On nodal level, a decrease in degree of the precentral gyrus is observed and an increase in degree of the left cerebellum part 6 and vermis part 6. As a higher degree indicates that the node is better connected, this seems contradictory to other literature that found that cerebellar dysfunction is common in MS [26]. However, functional reorganization to maintain high efficiency is a common phenomenon, which could lead to the generation of more connections in specific brain regions. The absence of significant differences for other network parameters in this study is likely due to the heterogeneity among MS patients, which can be caused by the random distribution of MS lesions or diffuse brain pathology. This implies that pathogenic effects are only observed when they are severe enough.

To compensate for the heterogeneity among MS patients and enhancing data-analysis sensitivity, lesion filling is often performed. Studies indicated that applying such a method enhances the reproducibility and reliability of GM and WM volume estimates [27–29]. This led to application of lesion filling for T1w structural graph theoretical network analysis in several studies. Due to the dark colour of the hypo intensity of MS lesions on T1w MRI, however, lesion intensities can be similar to GM, and therefore result in erroneous tissue segmentations. Since pathological changes represented by the lesions are omitted and a thorough evaluation of the effect of lesion filling has not yet been performed, the interpretability of the network analysis results after lesion-filling remains a matter of debate. Especially in graph theoretical network analysis, small changes in node intensity can have a substantial impact. For instance, if an affected hub is near a juxtacortical lesion, lesion filling might falsely insert extra GM voxels when “restoring” the structure, which might lead to erroneous results (see Figs. 3 and 4). In the current study, we also found a large number of artefacts arising from inaccurate lesion filling, especially in patients with a high number of lesions in the brain. The effects of minor artefacts, however, should be minimal, as they are considered to have no or negligible effects on tissue segmentation, and hence should hardly affect the calculation of the network parameters, if at all. However, for the substantial artefacts, the tissue segmentation is affected to some extent, but the effect on calculation of the network parameters might be limited as the lesions occur at random locations and as such the artefacts caused by lesion filling as well. The artefacts would therefore only cause false negative results, and thus result in less significant findings. Therefore, studies applying lesion filling should carefully assess the lesion-filled images for artefacts, especially for datasets containing a high number of brain lesions. A possible solution to optimize the accuracy of lesion-filling could be to include only WM voxels for lesion-filling of juxtacortical WM lesions.

Despite the imperfection of the lesion filling method, the 95% CIs of the network parameters derived from the lesion-filled T1w-based data were smaller than those derived from the original T1w-based data. Consequently, a considerable increase in discriminative power was obtained when lesion filling was applied. The heterogeneity in MS results in a high variation in the number of lesions, the size of lesions, and the location of lesions between patients. The applied graph theoretical method calculates the network parameters group-wise, so it is expected that a high degree of heterogeneity among subjects would affect the robustness of these calculations. This might also explain the large increase in the number of significant findings when lesion filling was applied, and thus enables detection of subtle differences with small datasets.

Among the network parameters derived from the original T1w MRI, differences were primarily detected on a global level. However, the original T1w MRI dataset generated multiple nodular parameters that were significantly different between groups (uncorrected) in several affected regions, but these effects did not survive FDR correction. In contrast, lesion filled T1w-based parameters showed significant differences on both a global and nodal level. This suggests that the results from lesion-filled MRI are not the result of the artefact introduced by the lesion filling method, since the differences are already present in the original data. Therefore, it seems likely that the variance in the original dataset was too high to reach statistical significance. By reducing the variance, lesion filling increases the statistical power. So, an alternative way to overcome the group heterogeneity could be by using large sample sizes without lesion filling.

Using the lesion-filled T1w dataset, we found an increase in global and local efficiency and a decrease in modularity in MS patients, as compared to HC. This is in agreement with findings of Fleischer (2017), and Kocevar (2016) [12, 14], and might suggest functional reorganization to maintain high cerebral efficiency. However, our findings of an increase in global and local efficiency and a decrease in modularity are in contrast to the results of other studies that found a decrease in global and local efficiency (He 2009, Shu 2011, Shu 2016, and Llufriu 2017) [8–10, 15]. Our study set-up is most similar to that of Kocevar, assessing different MS types with T1w MRI, whereas He and co-workers did not use healthy controls and investigated only RRMS with T1w MRI. The studies by Shu (2011), Shu (2016), and Llufriu used DTI to investigate RRMS, CIS, and the total MS population, respectively. The discrepancy between these studies illustrates the difficulties of comparing the graph theoretical network parameters derived from different structural networks, like WM connectivity assessed with DTI, and GM connectivity assessed with T1w.

Furthermore, our study shows that network parameters of RRMS patients in general deviate more from HC than those of PMS patients. This observation seems to be in agreement with the studies of Schoonheim and colleagues [30–32]. According to their hypothesis, functional reorganisation of the cerebral network takes place in MS patients as compensatory mechanism for structural damage, which is in agreement with our finding that a higher efficiency is found in RRMS patients than in HC. As a consequence, the network efficiency remains high enough for maintaining cognitive performances. However, there is a threshold for the functional reorganization capacity of the brain. When this threshold is reached, the brain is not able to fully compensate for the structural damage anymore, as suggested by the decreased efficiency observed in PMS compared to RRMS patients in this study. This is also supported by studies assessing axonal density in lesions that found a lower number of axons in progressive forms of MS compared to RRMS [33–35]. Thus, our results are in line with the findings of Fleischer, and Kocevar, and are supporting the Schoonheim hypothesis [12, 14, 30].

Future studies should thoroughly evaluate the accuracy of current lesion filling methods and evaluate the generation of artefacts on both T1w scans and tissue segmentations. Such a study would be able to determine the most optimal lesion filling method and indicate whether there is a need for further development of lesion filling methods. Until such a study is performed, no firm conclusions can be drawn regarding the optimal application of lesion filling. Nonetheless, our study clearly illustrates the positive effects lesion filling can have on the calculation of network parameters, despite the considerable number of artefacts generated, highlighting the need for cautious considerations before applying lesion filling.

In conclusion, we found that the application of lesion filling has reduced the variability and increased the sensitivity of the structural T1w network analysis. Although lesion filling is not perfect, we assume that application of lesion filling is especially important for studies with smaller sample sizes. In this study with a relatively small sample size, lesion filling indeed enabled graph theoretical network analysis to demonstrate that networks associated with cerebellum crus 2, cerebellum part 7b, and Heschl’s gyrus are affected in all types of MS patients, and that networks involving the supplementary motor area are only significantly affected in PMS patients.

## Supplementary Information

**Additional file 1.** Supplementary data showing network parameters that are significantly different per comparison for both grey matter fractions of original T1w scans and lesion filled T1w scans. Furthermore, an overview of the automated anatomical labelling (AAL) atlas regions of interest (ROIs), abbreviations, and lobular categorizations is provided.

## Data Availability

Due to privacy regulations, the clinical data collected in this study are not deposited in a public registry, but the data can be made available via a request to the corresponding author.

## Abbreviations

AAL: Automated anatomic labelling
BRAPH: Brain analysis using graph theory
CAL.L: Left calcarine fissure
CI: Confidence interval
CIS: Clinically isolated syndrome
CNS: Central nervous system
CSF: Cerebrospinal fluid
DCG.L: Left dorsolateral cingulate gyrus
DTI: Diffusion tensor imaging
EDSS: Expanded disability status scale
FDR: False discovery rate
GM: Grey matter
HC: Healthy control
LGA: Lesion growth algorithm
MNI: Montreal neurological institute
MRI: Magnetic resonance imaging
MS: Multiple sclerosis
PCL.R: Right paracentral lobule
PMS: Progressive MS
ROI: Region of interest
RRMS: Relapse-remitting MS
T1w: T1-weighted
WFU: Wake Forest University
WM: White matter
