# Supplementary material for: The effect of lesion filling on brain network analysis in multiple sclerosis using structural magnetic resonance imaging
Source: Insights Imaging. 2022 Mar 28;13:63. doi: 10.1186/s13244-022-01198-4 (PMC8960512; doi:10.1186/s13244-022-01198-4)
Supplement: Supplementary file 1 — Additional file 1. Supplementary data showing network parameters that are significantly different per comparison for both grey matter fractions of original T1w scans and lesion filled T1w scans. Furthermore, an overview of the automated anatomical labelling (AAL) atlas regions of interest (ROIs), abbreviations, and lobular categorizations is provided. [file 13244_2022_1198_MOESM1_ESM.docx]

**Appendix**

Table 1: Degree calculated from original T1w

| HC vs. PMS |  |  |  |  |  |  |  |  |
| --- | --- | --- | --- | --- | --- | --- | --- | --- |
|  | ROI | difference | p (2-tailed) | fdr (2-tailed) | HC | PMS | CI lower | CI upper |
| Frontal lobe |  |  |  |  |  |  |  |  |
|  | PreCG.L | -4 | 0.001 | 0.001 | 115 | 111 | -1.00 | 1.00 |
|  | PreCG.R | -5 | 0.001 | 0.001 | 115 | 110 | -2.00 | 2.00 |
| Posterior fossa |  |  |  |  |  |  |  |  |
|  | C6.L | 6 | 0.001 | 0.001 | 108 | 114 | -3.01 | 2.99 |
|  | V6 | 14 | 0.001 | 0.001 | 101 | 115 | -4.01 | 3.99 |

*solely the results of significantly different results that sustained FDR correction are presented

Table 2: Degree calculated from lesion filled T1

| HC vs. MS total |  |  |  |  |  |  |  |  |
| --- | --- | --- | --- | --- | --- | --- | --- | --- |
|  |  |  |  |  |  |  |  |  |
|  | ROI | difference | p (2-tailed) | fdr (2-tailed) | HC | MS total | CI lower | CI upper |
| Frontal |  |  |  |  |  |  |  |  |
|  | PreCG.L | 1 | 0.010 | 0.030 | 114 | 115 | 0.00 | 0.00 |
|  | PreCG.R | 1 | 0.006 | 0.030 | 114 | 115 | 0.00 | 0.00 |
|  | SFGdor.L | 2 | 0.005 | 0.030 | 113 | 115 | 0.00 | 1.00 |
|  | SFGdor.R | 3 | 0.001 | 0.030 | 112 | 115 | 0.00 | 1.00 |
|  | ORBsup.L | 12 | 0.004 | 0.030 | 103 | 115 | 0.02 | 2.00 |
|  | MFG.R | 2 | 0.003 | 0.030 | 113 | 115 | 0.00 | 1.00 |
|  | ORBmid.L | 4 | 0.004 | 0.030 | 111 | 115 | 0.00 | 1.00 |
|  | ORBmid.R | 6 | 0.002 | 0.030 | 109 | 115 | 0.00 | 1.00 |
|  | IFGtriang.R | 3 | 0.007 | 0.030 | 112 | 115 | 0.00 | 1.00 |
|  | ORBinf.L | 2 | 0.006 | 0.030 | 113 | 115 | 0.00 | 1.00 |
|  | ORBinf.R | 1 | 0.026 | 0.030 | 114 | 115 | 0.00 | 1.00 |
|  | ROL.R | 11 | 0.001 | 0.030 | 104 | 115 | 0.01 | 2.00 |
|  | SMA.L | 3 | 0.018 | 0.030 | 112 | 115 | 0.00 | 2.00 |
|  | SMA.R | 2 | 0.024 | 0.030 | 113 | 115 | 0.01 | 0.99 |
|  | OLF.R | 3 | 0.029 | 0.030 | 112 | 115 | 0.01 | 1.99 |
|  | ORBsupmed.L | 3 | 0.006 | 0.030 | 112 | 115 | 0.00 | 1.00 |
|  | ORBsupmed.R | 6 | 0.008 | 0.030 | 109 | 115 | 0.01 | 1.99 |
|  | REC.L | 4 | 0.018 | 0.030 | 111 | 115 | 0.00 | 1.99 |
|  | PCL.R | 10 | 0.001 | 0.030 | 105 | 115 | 0.00 | 2.99 |
| Insula and cingulate gyri |  |  |  |  |  |  |  |  |
|  | INS.R | 3 | 0.008 | 0.030 | 112 | 115 | 0.00 | 2.00 |
|  | DCG.L | 5 | 0.005 | 0.030 | 110 | 115 | 0.00 | 2.00 |
|  | DCG.R | 4 | 0.001 | 0.030 | 111 | 115 | 0.00 | 1.00 |
|  | PCG.L | 7 | 0.016 | 0.030 | 108 | 115 | 0.02 | 4.01 |
| Temporal |  |  |  |  |  |  |  |  |
|  | AMYG.L | 2 | 0.017 | 0.030 | 113 | 115 | 0.00 | 2.00 |
|  | AMYG.R | 8 | 0.017 | 0.030 | 107 | 115 | -0.01 | 4.99 |
|  | FFG.L | 1 | 0.027 | 0.030 | 114 | 115 | 0.00 | 1.00 |
|  | FFG.R | 5 | 0.006 | 0.030 | 110 | 115 | 0.01 | 1.00 |
|  | HES.L | 2 | 0.008 | 0.030 | 113 | 115 | 0.00 | 1.00 |
|  | HES.R | 7 | 0.001 | 0.030 | 108 | 115 | 0.01 | 0.99 |
|  | STG.L | 3 | 0.003 | 0.030 | 112 | 115 | 0.00 | 1.00 |
|  | STG.R | 1 | 0.010 | 0.030 | 114 | 115 | 0.00 | 1.00 |
|  | TPOsup.R | 3 | 0.005 | 0.030 | 112 | 115 | 0.00 | 1.00 |
|  | MTG.L | 6 | 0.001 | 0.030 | 109 | 115 | 0.00 | 0.00 |
|  | MTG.R | 2 | 0.006 | 0.030 | 113 | 115 | 0.00 | 1.00 |
|  | TPOmid.R | 3 | 0.030 | 0.030 | 112 | 115 | -0.01 | 2.99 |
|  | ITG.L | 8 | 0.002 | 0.030 | 107 | 115 | 0.01 | 0.99 |
|  | ITG.R | 2 | 0.003 | 0.030 | 113 | 115 | 0.00 | 1.00 |
| Occipital |  |  |  |  |  |  |  |  |
|  | CAL.L | 3 | 0.007 | 0.030 | 112 | 115 | 0.00 | 1.00 |
|  | CUN.L | 10 | 0.001 | 0.030 | 105 | 115 | 0.00 | 2.00 |
|  | LING.L | 0 | 0.023 | 0.030 | 115 | 115 | 0.00 | 0.00 |
|  | LING.R | 0 | 0.027 | 0.030 | 115 | 115 | 0.00 | 0.00 |
|  | SOG.R | 8 | 0.001 | 0.030 | 107 | 115 | 0.00 | 1.00 |
|  | MOG.L | 3 | 0.013 | 0.030 | 112 | 115 | 0.02 | 1.01 |
|  | MOG.R | 3 | 0.028 | 0.030 | 112 | 115 | 0.01 | 2.00 |
|  | IOG.R | 29 | 0.001 | 0.030 | 86 | 115 | 0.02 | 5.00 |
| Parietal |  |  |  |  |  |  |  |  |
|  | SPG.L | 3 | 0.017 | 0.030 | 112 | 115 | 0.01 | 1.99 |
|  | SPG.R | 8 | 0.005 | 0.030 | 107 | 115 | 0.01 | 3.00 |
|  | IPL.L | 1 | 0.018 | 0.030 | 114 | 115 | 0.00 | 1.00 |
|  | IPL.R | 6 | 0.004 | 0.030 | 109 | 115 | 0.01 | 2.00 |
|  | SMG.L | 2 | 0.006 | 0.030 | 113 | 115 | 0.00 | 1.00 |
|  | ANG.L | 5 | 0.003 | 0.030 | 110 | 115 | 0.01 | 1.00 |
|  | ANG.R | 1 | 0.028 | 0.030 | 114 | 115 | 0.00 | 1.00 |
|  | PCUN.L | 8 | 0.001 | 0.030 | 107 | 115 | 0.00 | 2.00 |
|  | PCUN.R | 5 | 0.002 | 0.030 | 110 | 115 | 0.00 | 2.00 |
| Central |  |  |  |  |  |  |  |  |
|  | PUT.L | 19 | 0.013 | 0.030 | 96 | 115 | 0.01 | 9.01 |
|  | THA.L | 6 | 0.008 | 0.030 | 109 | 115 | 0.01 | 3.00 |
|  | THA.R | 11 | 0.007 | 0.030 | 104 | 115 | -0.01 | 3.49 |
| Posterior fossa |  |  |  |  |  |  |  |  |
|  | CC1.R | 13 | 0.001 | 0.030 | 102 | 115 | 0.01 | 3.00 |
|  | CC2.L | 19 | 0.001 | 0.030 | 96 | 115 | 0.00 | 2.00 |
|  | CC2.R | 32 | 0.001 | 0.030 | 83 | 115 | 0.00 | 4.01 |
|  | C3.L | 4 | 0.010 | 0.030 | 111 | 115 | 0.01 | 2.00 |
|  | C3.R | 5 | 0.015 | 0.030 | 110 | 115 | -0.01 | 4.00 |
|  | C45.L | 8 | 0.004 | 0.030 | 107 | 115 | 0.00 | 2.99 |
|  | C45.R | 29 | 0.001 | 0.030 | 86 | 115 | 0.00 | 2.99 |
|  | C6.L | 10 | 0.001 | 0.030 | 105 | 115 | 0.00 | 3.00 |
|  | C6.R | 16 | 0.001 | 0.030 | 99 | 115 | 0.01 | 3.50 |
|  | C7b.R | 28 | 0.001 | 0.030 | 87 | 115 | 0.01 | 2.99 |
|  | C8.L | 2 | 0.008 | 0.030 | 113 | 115 | 0.00 | 1.00 |
|  | C8.R | 3 | 0.003 | 0.030 | 112 | 115 | 0.00 | 1.00 |
|  | C9.L | 4 | 0.021 | 0.030 | 111 | 115 | 0.00 | 3.00 |
|  | C9.R | 6 | 0.002 | 0.030 | 109 | 115 | 0.00 | 2.00 |
|  | V6 | 6 | 0.020 | 0.030 | 109 | 115 | -0.01 | 3.99 |
|  | V8 | 18 | 0.014 | 0.030 | 97 | 115 | -1.03 | 9.98 |
|  | V9 | 14 | 0.019 | 0.030 | 101 | 115 | 0.03 | 7.02 |
|  |  |  |  |  |  |  |  |  |
| HC vs. RRMS |  |  |  |  |  |  |  |  |
|  |  |  |  |  |  |  |  |  |
|  | ROI | difference | p (2-tailed) | fdr (2-tailed) | HC | RRMS | CI lower | CI upper |
| Frontal |  |  |  |  |  |  |  |  |
|  | PreCG.L | 1 | 0.016 | 0.021 | 114 | 115 | 0.00 | 1.00 |
|  | PreCG.R | 1 | 0.009 | 0.021 | 114 | 115 | 0.00 | 0.00 |
|  | SFGdor.L | 2 | 0.002 | 0.021 | 113 | 115 | 0.00 | 0.00 |
|  | SFGdor.R | 3 | 0.001 | 0.021 | 112 | 115 | 0.00 | 0.00 |
|  | ORBsup.L | 12 | 0.009 | 0.021 | 103 | 115 | 0.00 | 3.99 |
|  | MFG.R | 2 | 0.005 | 0.021 | 113 | 115 | 0.00 | 1.00 |
|  | ORBmid.L | 4 | 0.020 | 0.021 | 111 | 115 | -0.01 | 2.01 |
|  | ORBmid.R | 6 | 0.007 | 0.021 | 109 | 115 | -0.01 | 1.02 |
|  | ORBinf.L | 2 | 0.005 | 0.021 | 113 | 115 | -0.01 | 1.00 |
|  | ORBinf.R | 1 | 0.021 | 0.021 | 114 | 115 | 0.00 | 0.99 |
|  | ROL.R | 11 | 0.007 | 0.021 | 104 | 115 | 0.01 | 2.50 |
|  | SFGmed.R | 1 | 0.014 | 0.021 | 114 | 115 | 0.00 | 0.00 |
|  | ORBsupmed.L | 3 | 0.002 | 0.021 | 112 | 115 | 0.00 | 1.00 |
|  | PCL.R | 10 | 0.008 | 0.021 | 105 | 115 | -1.03 | 2.98 |
| Insula and cingulate gyri |  |  |  |  |  |  |  |  |
|  | DCG.L | 5 | 0.006 | 0.021 | 110 | 115 | -0.01 | 1.00 |
|  | DCG.R | 4 | 0.001 | 0.021 | 111 | 115 | 0.00 | 1.00 |
| Occipital |  |  |  |  |  |  |  |  |
|  | CAL.L | 3 | 0.017 | 0.021 | 112 | 115 | 0.00 | 2.00 |
|  | CUN.L | 10 | 0.003 | 0.021 | 105 | 115 | -0.01 | 1.99 |
|  | LING.L | 0 | 0.008 | 0.021 | 115 | 115 | 0.00 | 0.00 |
|  | LING.R | 0 | 0.016 | 0.021 | 115 | 115 | 0.00 | 0.00 |
|  | SOG.R | 8 | 0.002 | 0.021 | 107 | 115 | -0.01 | 2.00 |
|  | MOG.L | 3 | 0.013 | 0.021 | 112 | 115 | 0.00 | 1.00 |
|  | IOG.R | 29 | 0.001 | 0.021 | 86 | 115 | -0.99 | 7.00 |
| Parietal |  |  |  |  |  |  |  |  |
|  | SPG.R | 8 | 0.017 | 0.021 | 107 | 115 | -1.01 | 5.00 |
|  | IPL.L | 1 | 0.013 | 0.021 | 114 | 115 | 0.00 | 1.00 |
|  | IPL.R | 6 | 0.012 | 0.021 | 109 | 115 | -0.01 | 2.99 |
|  | SMG.L | 2 | 0.006 | 0.021 | 113 | 115 | 0.00 | 1.00 |
|  | ANG.L | 5 | 0.002 | 0.021 | 110 | 115 | 0.01 | 1.00 |
|  | PCUN.L | 8 | 0.009 | 0.021 | 107 | 115 | -0.02 | 2.98 |
|  | PCUN.R | 5 | 0.011 | 0.021 | 110 | 115 | -0.01 | 1.99 |
| Central |  |  |  |  |  |  |  |  |
|  | PUT.L | 19 | 0.001 | 0.021 | 96 | 115 | -0.02 | 4.02 |
|  | PUT.R | 18 | 0.017 | 0.021 | 97 | 115 | -0.99 | 9.99 |
|  | THA.L | 6 | 0.005 | 0.021 | 109 | 115 | 0.00 | 1.99 |
|  | THA.R | 11 | 0.001 | 0.021 | 104 | 115 | -1.00 | 3.00 |
| Temporal |  |  |  |  |  |  |  |  |
|  | HES.L | 2 | 0.012 | 0.021 | 113 | 115 | 0.00 | 1.00 |
|  | HES.R | 7 | 0.002 | 0.021 | 108 | 115 | -0.01 | 1.00 |
|  | STG.L | 3 | 0.011 | 0.021 | 112 | 115 | 0.00 | 1.00 |
|  | STG.R | 1 | 0.013 | 0.021 | 114 | 115 | 0.01 | 0.01 |
|  | TPOsup.R | 3 | 0.011 | 0.021 | 112 | 115 | 0.00 | 2.00 |
|  | MTG.L | 6 | 0.001 | 0.021 | 109 | 115 | 0.00 | 0.00 |
|  | MTG.R | 2 | 0.010 | 0.021 | 113 | 115 | 0.01 | 1.00 |
|  | ITG.L | 8 | 0.010 | 0.021 | 107 | 115 | -0.01 | 1.00 |
|  | ITG.R | 2 | 0.012 | 0.021 | 113 | 115 | 0.00 | 1.00 |
| Posterior fossa |  |  |  |  |  |  |  |  |
|  | CC1.R | 13 | 0.005 | 0.021 | 102 | 115 | -1.00 | 3.99 |
|  | CC2.L | 19 | 0.001 | 0.021 | 96 | 115 | -0.99 | 3.00 |
|  | CC2.R | 32 | 0.001 | 0.021 | 83 | 115 | -1.01 | 4.00 |
|  | C45.L | 8 | 0.010 | 0.021 | 107 | 115 | -0.99 | 3.50 |
|  | C45.R | 29 | 0.001 | 0.021 | 86 | 115 | -2.00 | 4.98 |
|  | C6.L | 10 | 0.002 | 0.021 | 105 | 115 | -1.00 | 3.01 |
|  | C6.R | 16 | 0.004 | 0.021 | 99 | 115 | -0.99 | 4.00 |
|  | C7b.L | 1 | 0.016 | 0.021 | 114 | 115 | 0.00 | 1.00 |
|  | C7b.R | 28 | 0.001 | 0.021 | 87 | 115 | -1.01 | 3.99 |
|  | C8.L | 2 | 0.018 | 0.021 | 113 | 115 | -0.01 | 0.99 |
|  | C8.R | 3 | 0.007 | 0.021 | 112 | 115 | 0.00 | 0.99 |
|  | C9.R | 6 | 0.017 | 0.021 | 109 | 115 | -1.00 | 4.00 |
|  | V7 | 5 | 0.019 | 0.021 | 110 | 115 | -0.01 | 2.99 |
|  | V8 | 18 | 0.020 | 0.021 | 97 | 115 | -3.03 | 10.03 |
|  | V9 | 14 | 0.011 | 0.021 | 101 | 115 | -1.01 | 5.00 |
|  |  |  |  |  |  |  |  |  |
| HC vs. PMS |  |  |  |  |  |  |  |  |
|  |  |  |  |  |  |  |  |  |
|  | ROI | difference | p (2-tailed) | fdr (2-tailed) | HC | PMS | CI lower | CI upper |
| Frontal |  |  |  |  |  |  |  |  |
|  | PreCG.L | 1 | 0.006 | 0.024 | 114 | 115 | 0.00 | 0.00 |
|  | PreCG.R | 1 | 0.007 | 0.024 | 114 | 115 | 0.00 | 0.00 |
|  | SFGdor.L | 2 | 0.007 | 0.024 | 113 | 115 | -1.00 | 1.00 |
|  | SFGdor.R | 3 | 0.001 | 0.024 | 112 | 115 | -1.00 | 1.00 |
|  | ORBsup.L | 12 | 0.001 | 0.024 | 103 | 115 | -2.00 | 1.01 |
|  | MFG.R | 2 | 0.008 | 0.024 | 113 | 115 | -1.00 | 1.00 |
|  | ORBmid.L | 4 | 0.006 | 0.024 | 111 | 115 | -1.00 | 1.00 |
|  | ORBmid.R | 6 | 0.005 | 0.024 | 109 | 115 | -2.01 | 1.99 |
|  | IFGtriang.R | 3 | 0.007 | 0.024 | 112 | 115 | -1.00 | 1.00 |
|  | ORBinf.L | 2 | 0.007 | 0.024 | 113 | 115 | -1.00 | 1.00 |
|  | ROL.R | 11 | 0.001 | 0.024 | 104 | 115 | -2.00 | 1.00 |
|  | SMA.L | -4 | 0.005 | 0.024 | 112 | 108 | -1.01 | 1.00 |
|  | OLF.R | 3 | 0.007 | 0.024 | 112 | 115 | -1.01 | 1.01 |
|  | SFGmed.R | 1 | 0.024 | 0.024 | 114 | 115 | -1.00 | 1.00 |
|  | ORBsupmed.L | 3 | 0.003 | 0.024 | 112 | 115 | -1.01 | 1.00 |
|  | ORBsupmed.R | 6 | 0.003 | 0.024 | 109 | 115 | -1.01 | 1.01 |
|  | REC.L | 4 | 0.010 | 0.024 | 111 | 115 | -1.01 | 0.99 |
| Insula and cingulate gyri |  |  |  |  |  |  |  |  |
|  | DCG.R | 3 | 0.007 | 0.024 | 111 | 114 | -1.01 | 1.00 |
| Temporal |  |  |  |  |  |  |  |  |
|  | AMYG.L | 2 | 0.024 | 0.024 | 113 | 115 | -1.00 | 1.00 |
|  | AMYG.R | 8 | 0.016 | 0.024 | 107 | 115 | -4.00 | 3.99 |
|  | FFG.R | 5 | 0.009 | 0.024 | 110 | 115 | -1.00 | 1.00 |
|  | HES.L | 2 | 0.008 | 0.024 | 113 | 115 | -1.00 | 0.00 |
|  | HES.R | 7 | 0.001 | 0.024 | 108 | 115 | 0.00 | 0.00 |
|  | STG.L | 3 | 0.001 | 0.024 | 112 | 115 | 0.00 | 0.00 |
|  | STG.R | 1 | 0.016 | 0.024 | 114 | 115 | -1.01 | -0.01 |
|  | TPOsup.R | 3 | 0.005 | 0.024 | 112 | 115 | -1.00 | 1.00 |
|  | MTG.L | 6 | 0.001 | 0.024 | 109 | 115 | -1.00 | 0.00 |
|  | MTG.R | 2 | 0.024 | 0.024 | 113 | 115 | -1.00 | 1.00 |
|  | TPOmid.L | 3 | 0.017 | 0.024 | 112 | 115 | -2.00 | 2.00 |
|  | TPOmid.R | 3 | 0.018 | 0.024 | 112 | 115 | -2.01 | 2.00 |
|  | ITG.L | 8 | 0.001 | 0.024 | 107 | 115 | 0.00 | 0.00 |
|  | ITG.R | 2 | 0.015 | 0.024 | 113 | 115 | -1.00 | 1.00 |
| Occipital |  |  |  |  |  |  |  |  |
|  | CAL.R | -3 | 0.023 | 0.024 | 115 | 112 | -1.00 | 1.00 |
|  | CUN.L | 10 | 0.001 | 0.024 | 105 | 115 | -2.00 | 1.99 |
|  | LING.L | 0 | 0.012 | 0.024 | 115 | 115 | 0.00 | 0.00 |
|  | LING.R | 0 | 0.024 | 0.024 | 115 | 115 | 0.00 | 0.00 |
|  | SOG.R | 8 | 0.001 | 0.024 | 107 | 115 | -2.50 | 2.00 |
|  | MOG.L | 3 | 0.004 | 0.024 | 112 | 115 | -1.00 | 0.00 |
|  | IOG.R | 29 | 0.015 | 0.024 | 86 | 115 | -10.99 | 8.97 |
| Parietal |  |  |  |  |  |  |  |  |
|  | SPG.R | 7 | 0.008 | 0.024 | 107 | 114 | -4.00 | 4.01 |
|  | IPL.R | 5 | 0.012 | 0.024 | 109 | 114 | -2.50 | 1.99 |
|  | SMG.L | 2 | 0.012 | 0.024 | 113 | 115 | -0.01 | -0.01 |
|  | ANG.L | 5 | 0.004 | 0.024 | 110 | 115 | -1.01 | 0.99 |
|  | ANG.R | 1 | 0.023 | 0.024 | 114 | 115 | -1.00 | 0.00 |
|  | PCUN.L | 7 | 0.003 | 0.024 | 107 | 114 | -2.99 | 2.99 |
|  | PCUN.R | 4 | 0.003 | 0.024 | 110 | 114 | -2.00 | 2.00 |
| Central |  |  |  |  |  |  |  |  |
|  | THA.L | 6 | 0.024 | 0.024 | 109 | 115 | -1.99 | 1.98 |
|  | THA.R | 11 | 0.019 | 0.024 | 104 | 115 | -3.00 | 3.50 |
| Posterior fossa |  |  |  |  |  |  |  |  |
|  | CC1.R | 13 | 0.001 | 0.024 | 102 | 115 | -3.99 | 3.01 |
|  | CC2.L | 18 | 0.001 | 0.024 | 96 | 114 | -4.00 | 4.00 |
|  | CC2.R | 28 | 0.003 | 0.024 | 83 | 111 | -6.99 | 7.03 |
|  | C3.L | 4 | 0.003 | 0.024 | 111 | 115 | -1.00 | 1.00 |
|  | C3.R | 5 | 0.009 | 0.024 | 110 | 115 | -3.01 | 2.00 |
|  | C45.L | 8 | 0.002 | 0.024 | 107 | 115 | -1.00 | 1.01 |
|  | C45.R | 28 | 0.001 | 0.024 | 86 | 114 | -3.02 | 3.01 |
|  | C6.L | 10 | 0.003 | 0.024 | 105 | 115 | -3.02 | 2.99 |
|  | C6.R | 16 | 0.001 | 0.024 | 99 | 115 | -5.01 | 5.00 |
|  | C7b.R | 28 | 0.001 | 0.024 | 87 | 115 | -5.00 | 2.98 |
|  | C8.L | 2 | 0.005 | 0.024 | 113 | 115 | -1.00 | 1.00 |
|  | C8.R | 3 | 0.006 | 0.024 | 112 | 115 | -2.00 | 1.00 |
|  | C9.L | 4 | 0.007 | 0.024 | 111 | 115 | -1.00 | 1.00 |
|  | C9.R | 6 | 0.003 | 0.024 | 109 | 115 | -2.01 | 1.00 |

*solely the results of significantly different results that sustained FDR correction are presented

Table 3: Strength calculated from lesion filled T1

| HC vs. MS total |  |  |  |  |  |  |  |  |
| --- | --- | --- | --- | --- | --- | --- | --- | --- |
|  |  |  |  |  |  |  |  |  |
|  | ROI | difference | p (2-tailed) | fdr (2-tailed) | HC | MS total | CI lower | CI upper |
| Frontal |  |  |  |  |  |  |  |  |
|  | MFG.R | 29.0 | 0.014 | 0.014 | 55.0 | 83.9 | -14.1 | 19.3 |
|  | ORBmid.L | 31.7 | 0.009 | 0.014 | 48.8 | 80.5 | -16.7 | 22.3 |
|  | ORBmid.R | 39.9 | 0.002 | 0.014 | 42.2 | 82.1 | -15.5 | 22.9 |
|  | IFGtriang.R | 30.9 | 0.006 | 0.014 | 50.6 | 81.6 | -15.1 | 21.4 |
|  | ORBinf.L | 31.5 | 0.005 | 0.014 | 54.6 | 86.1 | -13.8 | 21.3 |
|  | ORBinf.R | 25.2 | 0.012 | 0.014 | 59.4 | 84.6 | -12.3 | 19.7 |
|  | ROL.R | 41.7 | 0.005 | 0.014 | 36.8 | 78.5 | -18.5 | 25.1 |
|  | PCL.R | 38.1 | 0.013 | 0.014 | 31.0 | 69.1 | -21.8 | 27.6 |
| Insula and cingulate gyri |  |  |  |  |  |  |  |  |
|  | DCG.R | 33.5 | 0.008 | 0.014 | 46.6 | 80.1 | -15.3 | 23.1 |
| Temporal |  |  |  |  |  |  |  |  |
|  | AMYG.L | 40.4 | 0.002 | 0.014 | 35.7 | 76.0 | -16.5 | 22.3 |
|  | HES.R | 39.0 | 0.001 | 0.014 | 40.3 | 79.4 | -13.2 | 20.2 |
|  | STG.L | 27.8 | 0.014 | 0.014 | 51.6 | 79.4 | -15.2 | 21.2 |
|  | STG.R | 31.7 | 0.002 | 0.014 | 54.8 | 86.5 | -11.5 | 18.0 |
|  | TPOsup.L | 25.3 | 0.011 | 0.014 | 54.4 | 79.7 | -11.0 | 18.8 |
|  | TPOsup.R | 27.1 | 0.011 | 0.014 | 52.6 | 79.7 | -12.0 | 19.6 |
|  | MTG.L | 33.4 | 0.002 | 0.014 | 51.2 | 84.6 | -12.1 | 18.8 |
|  | MTG.R | 31.7 | 0.005 | 0.014 | 54.4 | 86.2 | -13.0 | 19.8 |
|  | ITG.R | 36.3 | 0.002 | 0.014 | 49.0 | 85.4 | -13.6 | 21.0 |
| Occipital |  |  |  |  |  |  |  |  |
|  | CAL.L | 39.3 | 0.003 | 0.014 | 44.6 | 83.9 | -15.9 | 23.2 |
|  | CUN.L | 47.3 | 0.001 | 0.014 | 37.6 | 84.9 | -16.5 | 26.4 |
|  | SOG.R | 43.0 | 0.003 | 0.014 | 35.4 | 78.4 | -17.9 | 24.6 |
| Parietal |  |  |  |  |  |  |  |  |
|  | PoCG.R | 31.8 | 0.002 | 0.014 | 53.2 | 85.0 | -12.2 | 19.0 |
|  | SPG.R | 42.5 | 0.008 | 0.014 | 33.3 | 75.8 | -21.5 | 29.5 |
|  | IPL.R | 36.9 | 0.010 | 0.014 | 39.7 | 76.5 | -18.8 | 25.4 |
|  | PCUN.L | 42.0 | 0.004 | 0.014 | 37.3 | 79.3 | -19.8 | 27.8 |
|  | PCUN.R | 41.6 | 0.003 | 0.014 | 39.6 | 81.3 | -17.4 | 24.3 |
| Posterior fossa |  |  |  |  |  |  |  |  |
|  | CC1.L | 33.0 | 0.004 | 0.014 | 47.2 | 80.2 | -13.1 | 20.7 |
|  | CC1.R | 53.7 | 0.001 | 0.014 | 25.0 | 78.7 | -18.5 | 27.4 |
|  | CC2.L | 52.2 | 0.001 | 0.014 | 27.6 | 79.8 | -15.5 | 22.9 |
|  | CC2.R | 55.4 | 0.001 | 0.014 | 18.9 | 74.3 | -21.0 | 30.4 |
|  | C45.R | 46.5 | 0.003 | 0.014 | 25.3 | 71.8 | -18.9 | 26.8 |
|  | C6.L | 38.9 | 0.006 | 0.014 | 38.9 | 77.8 | -17.1 | 25.3 |
|  | C6.R | 45.0 | 0.001 | 0.014 | 33.7 | 78.7 | -17.5 | 27.6 |
|  | C7b.L | 31.1 | 0.003 | 0.014 | 46.7 | 77.8 | -14.6 | 20.9 |
|  | C7b.R | 55.9 | 0.001 | 0.014 | 19.5 | 75.4 | -18.8 | 26.0 |
|  | C8.L | 33.5 | 0.007 | 0.014 | 42.4 | 75.9 | -16.1 | 23.0 |
|  | C8.R | 36.2 | 0.004 | 0.014 | 41.0 | 77.1 | -16.0 | 22.6 |
|  |  |  |  |  |  |  |  |  |
| HC vs. RRMS |  |  |  |  |  |  |  |  |
|  | ROI | difference | p (2-tailed) | fdr (2-tailed) | HC | RRMS | CI lower | CI upper |
| Frontal |  |  |  |  |  |  |  |  |
|  | ORBmid.R | 42.5 | 0.002 | 0.002 | 42.2 | 84.7 | -23.2 | 26.9 |
| Temporal |  |  |  |  |  |  |  |  |
|  | HES.R | 39.5 | 0.001 | 0.002 | 40.3 | 79.9 | -19.6 | 24.8 |
| Posterior fossa |  |  |  |  |  |  |  |  |
|  | CC2.L | 55.1 | 0.001 | 0.002 | 27.6 | 82.7 | -22.2 | 26.0 |
|  | CC2.R | 61.6 | 0.001 | 0.002 | 18.9 | 80.5 | -25.4 | 30.2 |
|  | C7b.R | 60.7 | 0.001 | 0.002 | 19.5 | 80.3 | -26.4 | 30.6 |

*solely the results of significantly different results that sustained FDR correction are presented

Table 4: Path length calculated from lesion filled T1

| HC vs. MS total |  |  |  |  |  |  |  |  |
| --- | --- | --- | --- | --- | --- | --- | --- | --- |
|  | ROI | difference | p (2-tailed) | fdr (2-tailed) | HC | MS total | CI lower | CI upper |
| Frontal |  |  |  |  |  |  |  |  |
|  | PreCG.L | -0.67 | 0.009 | 0.024 | 2.12 | 1.45 | -0.48 | 0.24 |
|  | PreCG.R | -0.71 | 0.007 | 0.024 | 2.16 | 1.45 | -0.43 | 0.23 |
|  | SFGdor.L | -0.74 | 0.007 | 0.024 | 2.21 | 1.47 | -0.45 | 0.23 |
|  | SFGdor.R | -0.80 | 0.011 | 0.024 | 2.27 | 1.47 | -0.48 | 0.26 |
|  | ORBsup.R | -0.97 | 0.012 | 0.024 | 2.49 | 1.52 | -0.70 | 0.38 |
|  | MFG.R | -0.79 | 0.005 | 0.024 | 2.21 | 1.42 | -0.46 | 0.23 |
|  | ORBmid.L | -0.87 | 0.006 | 0.024 | 2.37 | 1.50 | -0.58 | 0.32 |
|  | ORBmid.R | -1.09 | 0.001 | 0.024 | 2.55 | 1.46 | -0.56 | 0.31 |
|  | IFGoperc.R | -0.77 | 0.013 | 0.024 | 2.29 | 1.52 | -0.51 | 0.27 |
|  | IFGtriang.L | -0.59 | 0.021 | 0.024 | 2.06 | 1.47 | -0.45 | 0.27 |
|  | IFGtriang.R | -0.85 | 0.005 | 0.024 | 2.32 | 1.47 | -0.54 | 0.30 |
|  | ORBinf.L | -0.81 | 0.002 | 0.024 | 2.19 | 1.38 | -0.48 | 0.26 |
|  | ORBinf.R | -0.65 | 0.006 | 0.024 | 2.07 | 1.42 | -0.42 | 0.23 |
|  | ROL.L | -0.61 | 0.022 | 0.024 | 2.06 | 1.45 | -0.50 | 0.27 |
|  | ROL.R | -1.20 | 0.001 | 0.024 | 2.74 | 1.54 | -0.64 | 0.38 |
|  | SFGmed.L | -0.57 | 0.023 | 0.024 | 2.07 | 1.50 | -0.43 | 0.24 |
|  | ORBsupmed.L | -0.64 | 0.023 | 0.024 | 2.16 | 1.52 | -0.51 | 0.28 |
|  | PCL.R | -1.22 | 0.004 | 0.024 | 2.96 | 1.74 | -0.81 | 0.50 |
| Insula and cingulate gyri |  |  |  |  |  |  |  |  |
|  | INS.R | -0.85 | 0.012 | 0.024 | 2.37 | 1.52 | -0.65 | 0.34 |
|  | ACG.L | -0.76 | 0.018 | 0.024 | 2.35 | 1.59 | -0.57 | 0.33 |
|  | DCG.L | -0.95 | 0.023 | 0.024 | 2.57 | 1.62 | -0.76 | 0.42 |
|  | DCG.R | -1.03 | 0.005 | 0.024 | 2.51 | 1.48 | -0.55 | 0.29 |
|  | PCG.L | -1.31 | 0.019 | 0.024 | 3.03 | 1.71 | -0.97 | 0.67 |
| Temporal |  |  |  |  |  |  |  |  |
|  | HIP.L | -0.71 | 0.024 | 0.024 | 2.43 | 1.72 | -0.60 | 0.37 |
|  | AMYG.L | -1.40 | 0.001 | 0.024 | 2.96 | 1.55 | -0.62 | 0.35 |
|  | HES.L | -0.74 | 0.013 | 0.024 | 2.23 | 1.49 | -0.52 | 0.27 |
|  | HES.R | -1.16 | 0.001 | 0.024 | 2.65 | 1.49 | -0.51 | 0.25 |
|  | STG.L | -0.79 | 0.008 | 0.024 | 2.29 | 1.50 | -0.51 | 0.28 |
|  | STG.R | -0.81 | 0.001 | 0.024 | 2.19 | 1.38 | -0.38 | 0.20 |
|  | TPOsup.L | -0.67 | 0.005 | 0.024 | 2.18 | 1.51 | -0.39 | 0.22 |
|  | TPOsup.R | -0.73 | 0.005 | 0.024 | 2.24 | 1.50 | -0.45 | 0.24 |
|  | MTG.L | -0.86 | 0.001 | 0.024 | 2.27 | 1.40 | -0.40 | 0.21 |
|  | MTG.R | -0.84 | 0.001 | 0.024 | 2.22 | 1.38 | -0.46 | 0.24 |
|  | TPOmid.R | -0.96 | 0.021 | 0.024 | 2.64 | 1.69 | -0.75 | 0.47 |
|  | ITG.L | -0.87 | 0.013 | 0.024 | 2.36 | 1.49 | -0.64 | 0.35 |
|  | ITG.R | -0.98 | 0.001 | 0.024 | 2.37 | 1.40 | -0.47 | 0.25 |
| Occipital |  |  |  |  |  |  |  |  |
|  | CAL.L | -1.11 | 0.001 | 0.024 | 2.53 | 1.42 | -0.54 | 0.29 |
|  | CUN.L | -1.36 | 0.001 | 0.024 | 2.75 | 1.39 | -0.57 | 0.34 |
|  | LING.L | -0.60 | 0.019 | 0.024 | 2.05 | 1.45 | -0.45 | 0.24 |
|  | LING.R | -0.61 | 0.023 | 0.024 | 2.04 | 1.44 | -0.46 | 0.24 |
|  | SOG.R | -1.40 | 0.001 | 0.024 | 2.91 | 1.51 | -0.67 | 0.38 |
|  | IOG.R | -1.50 | 0.008 | 0.024 | 3.14 | 1.64 | -1.03 | 0.73 |
| Parietal |  |  |  |  |  |  |  |  |
|  | PoCG.L | -0.61 | 0.009 | 0.024 | 2.06 | 1.45 | -0.39 | 0.21 |
|  | PoCG.R | -0.86 | 0.001 | 0.024 | 2.26 | 1.40 | -0.40 | 0.21 |
|  | SPG.R | -1.36 | 0.004 | 0.024 | 2.95 | 1.58 | -0.82 | 0.51 |
|  | IPL.L | -0.69 | 0.013 | 0.024 | 2.16 | 1.47 | -0.46 | 0.25 |
|  | IPL.R | -1.10 | 0.012 | 0.024 | 2.68 | 1.58 | -0.69 | 0.40 |
|  | SMG.L | -0.82 | 0.009 | 0.024 | 2.29 | 1.46 | -0.54 | 0.28 |
|  | SMG.R | -0.80 | 0.011 | 0.024 | 2.23 | 1.43 | -0.54 | 0.29 |
|  | ANG.R | -0.72 | 0.016 | 0.024 | 2.18 | 1.46 | -0.52 | 0.27 |
|  | PCUN.L | -1.27 | 0.004 | 0.024 | 2.77 | 1.50 | -0.71 | 0.38 |
|  | PCUN.R | -1.21 | 0.002 | 0.024 | 2.67 | 1.46 | -0.60 | 0.32 |
| Posterior fossa |  |  |  |  |  |  |  |  |
|  | CC1.L | -0.92 | 0.003 | 0.024 | 2.40 | 1.48 | -0.51 | 0.26 |
|  | CC1.R | -1.78 | 0.001 | 0.024 | 3.29 | 1.51 | -0.73 | 0.40 |
|  | CC2.L | -1.65 | 0.001 | 0.024 | 3.15 | 1.50 | -0.56 | 0.31 |
|  | CC2.R | -2.10 | 0.001 | 0.024 | 3.72 | 1.62 | -0.85 | 0.49 |
|  | C3.R | -1.01 | 0.012 | 0.024 | 2.74 | 1.73 | -0.74 | 0.45 |
|  | C45.L | -0.99 | 0.007 | 0.024 | 2.75 | 1.76 | -0.71 | 0.42 |
|  | C45.R | -1.62 | 0.001 | 0.024 | 3.28 | 1.66 | -0.72 | 0.43 |
|  | C6.L | -1.24 | 0.002 | 0.024 | 2.77 | 1.53 | -0.61 | 0.36 |
|  | C6.R | -1.37 | 0.001 | 0.024 | 2.88 | 1.51 | -0.68 | 0.38 |
|  | C7b.L | -0.94 | 0.002 | 0.024 | 2.48 | 1.53 | -0.50 | 0.27 |
|  | C7b.R | -2.06 | 0.001 | 0.024 | 3.66 | 1.59 | -0.72 | 0.40 |
|  | C8.L | -1.05 | 0.005 | 0.024 | 2.60 | 1.55 | -0.61 | 0.33 |
|  | C8.R | -1.07 | 0.002 | 0.024 | 2.61 | 1.54 | -0.56 | 0.32 |
|  | C9.L | -0.96 | 0.023 | 0.024 | 2.83 | 1.87 | -0.78 | 0.49 |
|  | C9.R | -1.05 | 0.007 | 0.024 | 2.77 | 1.72 | -0.74 | 0.43 |
|  |  |  |  |  |  |  |  |  |
| HC vs. RRMS |  |  |  |  |  |  |  |  |
|  | ROI | difference | p (2-tailed) | fdr (2-tailed) | HC | RRMS | CI lower | CI upper |
| Frontal |  |  |  |  |  |  |  |  |
|  | ORBmid.R | -1.15 | 0.005 | 0.009 | 2.55 | 1.40 | -0.69 | 0.53 |
|  | ROL.R | -1.29 | 0.005 | 0.009 | 2.74 | 1.44 | -0.84 | 0.64 |
|  | PCL.R | -1.41 | 0.004 | 0.009 | 2.96 | 1.55 | -0.87 | 0.71 |
| Insula and cingulate gyri |  |  |  |  |  |  |  |  |
|  | DCG.L | -1.13 | 0.006 | 0.009 | 2.57 | 1.45 | -0.70 | 0.50 |
|  | DCG.R | -1.12 | 0.005 | 0.009 | 2.51 | 1.39 | -0.67 | 0.48 |
| Temporal |  |  |  |  |  |  |  |  |
|  | AMYG.L | -1.42 | 0.002 | 0.009 | 2.96 | 1.54 | -0.79 | 0.63 |
|  | HES.R | -1.17 | 0.001 | 0.009 | 2.65 | 1.49 | -0.62 | 0.46 |
|  | STG.R | -0.86 | 0.005 | 0.009 | 2.19 | 1.33 | -0.52 | 0.38 |
|  | MTG.L | -0.93 | 0.003 | 0.009 | 2.27 | 1.34 | -0.51 | 0.36 |
|  | MTG.R | -0.89 | 0.005 | 0.009 | 2.22 | 1.33 | -0.57 | 0.38 |
|  | ITG.R | -1.02 | 0.004 | 0.009 | 2.37 | 1.35 | -0.61 | 0.43 |
| Occipital |  |  |  |  |  |  |  |  |
|  | CAL.L | -1.17 | 0.006 | 0.009 | 2.53 | 1.36 | -0.71 | 0.53 |
|  | CUN.L | -1.38 | 0.005 | 0.009 | 2.75 | 1.37 | -0.80 | 0.61 |
|  | SOG.R | -1.46 | 0.002 | 0.009 | 2.91 | 1.45 | -0.79 | 0.60 |
|  | IOG.R | -1.56 | 0.006 | 0.009 | 3.14 | 1.58 | -1.03 | 0.94 |
| Parietal |  |  |  |  |  |  |  |  |
|  | PoCG.R | -0.88 | 0.008 | 0.009 | 2.26 | 1.39 | -0.52 | 0.36 |
|  | PCUN.L | -1.27 | 0.009 | 0.009 | 2.77 | 1.50 | -0.93 | 0.68 |
|  | PCUN.R | -1.23 | 0.009 | 0.009 | 2.67 | 1.44 | -0.83 | 0.62 |
| Posterior fossa |  |  |  |  |  |  |  |  |
|  | CC1.R | -1.75 | 0.001 | 0.009 | 3.29 | 1.54 | -0.93 | 0.72 |
|  | CC2.L | -1.72 | 0.001 | 0.009 | 3.15 | 1.43 | -0.71 | 0.54 |
|  | CC2.R | -2.25 | 0.001 | 0.009 | 3.72 | 1.47 | -0.83 | 0.64 |
|  | C45.R | -1.72 | 0.001 | 0.009 | 3.28 | 1.55 | -0.88 | 0.73 |
|  | C6.L | -1.27 | 0.004 | 0.009 | 2.77 | 1.50 | -0.78 | 0.55 |
|  | C6.R | -1.44 | 0.003 | 0.009 | 2.88 | 1.44 | -0.80 | 0.61 |
|  | C7b.R | -2.17 | 0.001 | 0.009 | 3.66 | 1.48 | -0.87 | 0.68 |
|  | C8.R | -1.08 | 0.007 | 0.009 | 2.61 | 1.53 | -0.78 | 0.52 |
|  |  |  |  |  |  |  |  |  |
| HC vs. PMS |  |  |  |  |  |  |  |  |
|  | ROI | difference | p (2-tailed) | fdr (2-tailed) | HC | PMS | CI lower | CI upper |
| Occipital |  |  |  |  |  |  |  |  |
|  | CUN.L | -1.25 | 0.003 | 0.003 | 2.75 | 1.50 | -0.67 | 0.63 |
| Parietal |  |  |  |  |  |  |  |  |
|  | PoCG.R | -0.74 | 0.001 | 0.003 | 2.26 | 1.52 | -0.34 | 0.33 |
| Temporal |  |  |  |  |  |  |  |  |
|  | HES.R | -1.03 | 0.001 | 0.003 | 2.65 | 1.63 | -0.46 | 0.44 |
|  | TPOsup.R | -0.69 | 0.001 | 0.003 | 2.24 | 1.55 | -0.37 | 0.33 |
| Posterior fossa |  |  |  |  |  |  |  |  |
|  | CC1.R | -1.60 | 0.001 | 0.003 | 3.29 | 1.69 | -0.84 | 0.74 |
|  | CC2.L | -1.41 | 0.001 | 0.003 | 3.15 | 1.74 | -0.70 | 0.63 |
|  | C7b.L | -0.84 | 0.001 | 0.003 | 2.48 | 1.63 | -0.38 | 0.35 |
|  | C7b.R | -1.91 | 0.001 | 0.003 | 3.66 | 1.74 | -0.86 | 0.82 |
|  | C8.R | -0.89 | 0.003 | 0.003 | 2.61 | 1.73 | -0.49 | 0.47 |

*solely the results of significantly different results that sustained FDR correction are presented

Table 5: Global efficiency calculated from lesion filled T1

| HC vs. MS total |  |  |  |  |  |  |  |  |
| --- | --- | --- | --- | --- | --- | --- | --- | --- |
|  | ROI | difference | p (2-tailed) | fdr (2-tailed) | HC | MS total | CI lower | CI upper |
| Posterior fossa |  |  |  |  |  |  |  |  |
|  | CC1.R | 0.36 | 0.001 | 0.001 | 0.32 | 0.68 | -0.16 | 0.22 |
|  | CC2.L | 0.36 | 0.001 | 0.001 | 0.34 | 0.69 | -0.13 | 0.18 |
|  | C7b.R | 0.37 | 0.001 | 0.001 | 0.29 | 0.66 | -0.16 | 0.21 |
|  |  |  |  |  |  |  |  |  |
| HC vs. RRMS |  |  |  |  |  |  |  |  |
|  | ROI | difference | p (2-tailed) | fdr (2-tailed) | HC | RRMS | CI lower | CI upper |
| Posterior fossa |  |  |  |  |  |  |  |  |
|  | CC2.L | 0.38 | 0.001 | 0.001 | 0.34 | 0.72 | -0.19 | 0.21 |
|  | CC2.R | 0.42 | 0.001 | 0.001 | 0.28 | 0.70 | -0.22 | 0.22 |
|  | C7b.R | 0.41 | 0.001 | 0.001 | 0.29 | 0.70 | -0.22 | 0.23 |

*solely the results of significantly different results that sustained FDR correction are presented

Table 6: Clustering coefficient calculated from lesion filled T1

| HC vs. MS total |  |  |  |  |  |  |  |  |
| --- | --- | --- | --- | --- | --- | --- | --- | --- |
|  | ROI | difference | p (2-tailed) | fdr (2-tailed) | HC | MS total | CI lower | CI upper |
| Frontal |  |  |  |  |  |  |  |  |
|  | PreCG.L | 0.25 | 0.016 | 0.024 | 0.44 | 0.68 | -0.12 | 0.19 |
|  | PreCG.R | 0.25 | 0.016 | 0.024 | 0.43 | 0.68 | -0.12 | 0.19 |
|  | SFGdor.L | 0.26 | 0.011 | 0.024 | 0.42 | 0.68 | -0.12 | 0.18 |
|  | SFGdor.R | 0.26 | 0.013 | 0.024 | 0.41 | 0.68 | -0.12 | 0.20 |
|  | ORBsup.R | 0.30 | 0.011 | 0.024 | 0.36 | 0.66 | -0.13 | 0.22 |
|  | MFG.R | 0.27 | 0.010 | 0.024 | 0.42 | 0.69 | -0.12 | 0.18 |
|  | ORBmid.L | 0.29 | 0.007 | 0.024 | 0.39 | 0.67 | -0.13 | 0.20 |
|  | ORBmid.R | 0.33 | 0.002 | 0.024 | 0.35 | 0.68 | -0.13 | 0.19 |
|  | IFGoperc.R | 0.27 | 0.012 | 0.024 | 0.40 | 0.66 | -0.12 | 0.19 |
|  | IFGtriang.L | 0.24 | 0.014 | 0.024 | 0.43 | 0.68 | -0.12 | 0.18 |
|  | IFGtriang.R | 0.29 | 0.009 | 0.024 | 0.39 | 0.68 | -0.12 | 0.19 |
|  | ORBinf.L | 0.28 | 0.006 | 0.024 | 0.42 | 0.70 | -0.11 | 0.19 |
|  | ORBinf.R | 0.26 | 0.010 | 0.024 | 0.44 | 0.70 | -0.10 | 0.18 |
|  | ROL.L | 0.24 | 0.021 | 0.024 | 0.44 | 0.68 | -0.12 | 0.19 |
|  | ROL.R | 0.33 | 0.005 | 0.024 | 0.33 | 0.66 | -0.14 | 0.21 |
|  | SFGmed.L | 0.24 | 0.021 | 0.024 | 0.44 | 0.67 | -0.11 | 0.19 |
|  | PCL.R | 0.32 | 0.022 | 0.024 | 0.28 | 0.61 | -0.16 | 0.25 |
| Insula and cingulate gyri |  |  |  |  |  |  |  |  |
|  | INS.L | 0.24 | 0.014 | 0.024 | 0.45 | 0.68 | -0.11 | 0.18 |
|  | INS.R | 0.28 | 0.006 | 0.024 | 0.38 | 0.67 | -0.13 | 0.19 |
|  | ACG.L | 0.26 | 0.016 | 0.024 | 0.39 | 0.64 | -0.13 | 0.20 |
|  | DCG.R | 0.29 | 0.009 | 0.024 | 0.38 | 0.67 | -0.13 | 0.20 |
|  | PCG.L | 0.31 | 0.020 | 0.024 | 0.30 | 0.61 | -0.17 | 0.24 |
| Temporal |  |  |  |  |  |  |  |  |
|  | AMYG.L | 0.34 | 0.004 | 0.024 | 0.31 | 0.65 | -0.13 | 0.19 |
|  | FFG.L | 0.26 | 0.023 | 0.024 | 0.43 | 0.68 | -0.12 | 0.20 |
|  | HES.L | 0.26 | 0.015 | 0.024 | 0.41 | 0.67 | -0.12 | 0.19 |
|  | HES.R | 0.31 | 0.004 | 0.024 | 0.35 | 0.67 | -0.11 | 0.18 |
|  | STG.L | 0.26 | 0.010 | 0.024 | 0.40 | 0.67 | -0.12 | 0.18 |
|  | STG.R | 0.30 | 0.002 | 0.024 | 0.41 | 0.71 | -0.10 | 0.17 |
|  | TPOsup.L | 0.25 | 0.008 | 0.024 | 0.42 | 0.67 | -0.11 | 0.17 |
|  | TPOsup.R | 0.26 | 0.010 | 0.024 | 0.41 | 0.67 | -0.11 | 0.18 |
|  | MTG.L | 0.29 | 0.003 | 0.024 | 0.40 | 0.70 | -0.10 | 0.17 |
|  | MTG.R | 0.29 | 0.005 | 0.024 | 0.42 | 0.70 | -0.11 | 0.18 |
|  | ITG.L | 0.27 | 0.020 | 0.024 | 0.40 | 0.67 | -0.14 | 0.21 |
|  | ITG.R | 0.32 | 0.002 | 0.024 | 0.38 | 0.70 | -0.11 | 0.19 |
| Occipital |  |  |  |  |  |  |  |  |
|  | CAL.L | 0.33 | 0.004 | 0.024 | 0.36 | 0.69 | -0.13 | 0.20 |
|  | CUN.L | 0.36 | 0.003 | 0.024 | 0.34 | 0.70 | -0.14 | 0.22 |
|  | LING.L | 0.24 | 0.024 | 0.024 | 0.44 | 0.68 | -0.12 | 0.19 |
|  | SOG.R | 0.34 | 0.002 | 0.024 | 0.32 | 0.66 | -0.14 | 0.21 |
| Parietal |  |  |  |  |  |  |  |  |
|  | PoCG.L | 0.24 | 0.018 | 0.024 | 0.45 | 0.68 | -0.11 | 0.18 |
|  | PoCG.R | 0.30 | 0.002 | 0.024 | 0.40 | 0.70 | -0.11 | 0.18 |
|  | SPG.R | 0.35 | 0.009 | 0.024 | 0.29 | 0.65 | -0.16 | 0.23 |
|  | IPL.L | 0.25 | 0.013 | 0.024 | 0.42 | 0.68 | -0.12 | 0.19 |
|  | IPL.R | 0.31 | 0.012 | 0.024 | 0.34 | 0.65 | -0.15 | 0.21 |
|  | SMG.L | 0.27 | 0.011 | 0.024 | 0.41 | 0.68 | -0.12 | 0.20 |
|  | SMG.R | 0.29 | 0.009 | 0.024 | 0.40 | 0.69 | -0.13 | 0.20 |
|  | ANG.R | 0.26 | 0.022 | 0.024 | 0.43 | 0.68 | -0.13 | 0.20 |
|  | PCUN.L | 0.35 | 0.006 | 0.024 | 0.32 | 0.67 | -0.15 | 0.22 |
|  | PCUN.R | 0.34 | 0.004 | 0.024 | 0.33 | 0.68 | -0.13 | 0.20 |
| Posterior fossa |  |  |  |  |  |  |  |  |
|  | CC1.L | 0.31 | 0.005 | 0.024 | 0.36 | 0.67 | -0.12 | 0.19 |
|  | CC1.R | 0.41 | 0.001 | 0.024 | 0.25 | 0.66 | -0.15 | 0.21 |
|  | CC2.L | 0.39 | 0.001 | 0.024 | 0.28 | 0.67 | -0.12 | 0.19 |
|  | CC2.R | 0.40 | 0.002 | 0.024 | 0.24 | 0.64 | -0.15 | 0.23 |
|  | C3.R | 0.29 | 0.017 | 0.024 | 0.32 | 0.61 | -0.15 | 0.22 |
|  | C45.R | 0.35 | 0.004 | 0.024 | 0.27 | 0.62 | -0.14 | 0.21 |
|  | C6.L | 0.32 | 0.004 | 0.024 | 0.33 | 0.66 | -0.14 | 0.20 |
|  | C6.R | 0.35 | 0.003 | 0.024 | 0.31 | 0.66 | -0.15 | 0.21 |
|  | C7b.L | 0.29 | 0.004 | 0.024 | 0.37 | 0.66 | -0.11 | 0.18 |
|  | C7b.R | 0.41 | 0.001 | 0.024 | 0.24 | 0.64 | -0.13 | 0.21 |
|  | C8.L | 0.31 | 0.004 | 0.024 | 0.34 | 0.65 | -0.12 | 0.20 |
|  | C8.R | 0.32 | 0.003 | 0.024 | 0.33 | 0.65 | -0.12 | 0.20 |
|  | C9.R | 0.28 | 0.018 | 0.024 | 0.32 | 0.60 | -0.15 | 0.20 |
|  | C10.R | 0.25 | 0.022 | 0.024 | 0.38 | 0.62 | -0.13 | 0.21 |
|  |  |  |  |  |  |  |  |  |
| HC vs. RRMS |  |  |  |  |  |  |  |  |
|  | ROI | difference | p (2-tailed) | fdr (2-tailed) | HC | RRMS | CI lower | CI upper |
| Posterior fossa |  |  |  |  |  |  |  |  |
|  | CC1.R | 0.41 | 0.001 | 0.001 | 0.25 | 0.66 | -0.21 | 0.26 |
|  | CC2.R | 0.44 | 0.001 | 0.001 | 0.24 | 0.68 | -0.21 | 0.24 |
|  | C7b.R | 0.44 | 0.001 | 0.001 | 0.24 | 0.68 | -0.20 | 0.24 |

*solely the results of significantly different results that sustained FDR correction are presented

Table 7: AAL atlas ROI abbreviations and full names

|  | AAL label | Abbreviation | Full name |
| --- | --- | --- | --- |
| Temporal lobe |  |  |  |
|  | 37 | HIP.L | Left Hippocampus |
|  | 38 | HIP.R | Right Hippocampus |
|  | 39 | PHG.L | Left Parahippocampal gyrus |
|  | 40 | PHG.R | Right Parahippocampal gyrus |
|  | 41 | AMYG.L | Left Amygdala |
|  | 42 | AMYG.R | Right Amygdala |
|  | 55 | FFG.L | Left Fusiform gyrus |
|  | 56 | FFG.R | Right Fusiform gyrus |
|  | 79 | HES.L | Left Heschl gyrus |
|  | 80 | HES.R | Right Heschl gyrus |
|  | 81 | STG.L | Left Superior temporal gyrus |
|  | 82 | STG.R | Right Superior temporal gyrus |
|  | 83 | TPOsup.L | Left Temporal pole: superior temporal gyrus |
|  | 84 | TPOsup.R | Right Temporal pole: superior temporal gyrus |
|  | 85 | MTG.L | Left Middle temporal gyrus |
|  | 86 | MTG.R | Right Middle temporal gyrus |
|  | 87 | TPOmid.L | Left Temporal pole: middle temporal gyrus |
|  | 88 | TPOmid.R | Right Temporal pole: middle temporal gyrus |
|  | 89 | ITG.L | Left Inferior temporal gyrus |
|  | 90 | ITG.R | Right Inferior temporal gyrus |
| Frontal lobe |  |  |  |
|  | 1 | PreCG.L | Left Precental gyrus |
|  | 2 | PreCG.R | Right Precental gyrus |
|  | 3 | SFGdor.L | Left Superior frontal gyrus, dorsolateral |
|  | 4 | SFGdor.R | Right Superior frontal gyrus, dorsolateral |
|  | 5 | ORBsup.L | Left Superior frontal gyrus, orbital part |
|  | 6 | ORBsup.R | Right Superior frontal gyrus, orbital part |
|  | 7 | MFG.L | Left Middle frontal gyrus |
|  | 8 | MFG.R | Right Middle frontal gyrus |
|  | 9 | ORBmid.L | Left Middle frontal gyrus, orbital part |
|  | 10 | ORBmid.R | Right Middle frontal gyrus, orbital part |
|  | 11 | IFGoperc.L | Left Inferior frontal gyrus, opercular part |
|  | 12 | IFGoperc.R | Right Inferior frontal gyrus, opercular part |
|  | 13 | IFGtriang.L | Left Inferior frontal gyrus, triangular part |
|  | 14 | IFGtriang.R | Right Inferior frontal gyrus, triangular part |
|  | 15 | ORBinf.L | Left Inferior frontal gyrus, orbital part |
|  | 16 | ORBinf.R | Right Inferior frontal gyrus, orbital part |
|  | 17 | ROL.L | Left Rolandic operculum |
|  | 18 | ROL.R | Right Rolandic operculum |
|  | 19 | SMA.L | Left Supplementary motor area |
|  | 20 | SMA.R | Right Supplementary motor area |
|  | 21 | OLF.L | Left Olfactory cortex |
|  | 22 | OLF.R | Right Olfactory cortex |
|  | 23 | SFGmed.L | Left Superior frontal gyrus, medial |
|  | 24 | SFGmed.R | Right Superior frontal gyrus, medial |
|  | 25 | ORBsupmed.L | Left Superior frontal gyrus, medial orbital |
|  | 26 | ORBsupmed.R | Right Superior frontal gyrus, medial orbital |
|  | 27 | REC.L | Left Gyrus rectus |
|  | 28 | REC.R | Right Gyrus rectus |
|  | 69 | PCL.L | Left Paracentral lobule |
|  | 70 | PCL.R | Right Paracentral lobule |
| Occipital lobe |  |  |  |
|  | 43 | CAL.L | Left Calcarine fissure and surrounding cortex |
|  | 44 | CAL.R | Right Calcarine fissure and surrounding cortex |
|  | 45 | CUN.L | Left Cuneus |
|  | 46 | CUN.R | Right Cuneus |
|  | 47 | LING.L | Left Lingual gyrus |
|  | 48 | LING.R | Right Lingual gyrus |
|  | 49 | SOG.L | Left Superior occipital gyrus |
|  | 50 | SOG.R | Right Superior occipital gyrus |
|  | 51 | MOG.L | Left Middle occipital gyrus |
|  | 52 | MOG.R | Right Middle occipital gyrus |
|  | 53 | IOG.L | Left Inferior occipital gyrus |
|  | 54 | IOG.R | Right Inferior occipital gyrus |
| Parietal lobe |  |  |  |
|  | 57 | PoCG.L | Left Postcentral gyrus |
|  | 58 | PoCG.R | Right Postcentral gyrus |
|  | 59 | SPG.L | Left Superior parietal gyrus |
|  | 60 | SPG.R | Right Superior parietal gyrus |
|  | 61 | IPL.L | Left Inferior parietal, but supramarginal and angular gyri |
|  | 62 | IPL.R | Right Inferior parietal, but supramarginal and angular gyri |
|  | 63 | SMG.L | Left Supramarginal gyrus |
|  | 64 | SMG.R | Right Supramarginal gyrus |
|  | 65 | ANG.L | Left Angular gyrus |
|  | 66 | ANG.R | Right Angular gyrus |
|  | 67 | PCUN.L | Left Precuneus |
|  | 68 | PCUN.R | Right Precuneus |
| Insula and Cingulate gyri |  |  |  |
|  | 29 | INS.L | Left Insula |
|  | 30 | INS.R | Right Insula |
|  | 31 | ACG.L | Left Anterior cingulate and paracingulate gyri |
|  | 32 | ACG.R | Right Anterior cingulate and paracingulate gyri |
|  | 33 | DCG.L | Left Median cingulate and paracingulate gyri |
|  | 34 | DCG.R | Right Median cingulate and paracingulate gyri |
|  | 35 | PCG.L | Left Posterior cingulate gyrus |
|  | 36 | PCG.R | Right Posterior cingulate gyrus |
| Central structures |  |  |  |
|  | 71 | CAU.L | Left Caudate nucleus |
|  | 72 | CAU.R | Right Caudate nucleus |
|  | 73 | PUT.L | Left Lenticular nucleus, putamen |
|  | 74 | PUT.R | Right Lenticular nucleus, putamen |
|  | 75 | PAL.L | Left Lenticular nucleus, pallidum |
|  | 76 | PAL.R | Right Lenticular nucleus, pallidum |
|  | 77 | THA.L | Left Thalamus |
|  | 78 | THA.R | Thalamus |
| Posterior fossa |  |  |  |
|  | 91 | CC1.L | Left Cerebellum crus part 1 |
|  | 92 | CC1.R | Right Cerebellum crus part 1 |
|  | 93 | CC2.L | Left Cerebellum crus part 2 |
|  | 94 | CC2.R | Right Cerebellum crus part 2 |
|  | 95 | C3.L | Left Cerebellum part 3 |
|  | 96 | C3.R | Right Cerebellum part 3 |
|  | 97 | C45.L | Left Cerebellum part 4 & 5 |
|  | 98 | C45.R | Right Cerebellum part 4 & 5 |
|  | 99 | C6.L | Left Cerebellum part 6 |
|  | 100 | C6.R | Right Cerebellum part 6 |
|  | 101 | C7b.L | Left Cerebellum part 7b |
|  | 102 | C7b.R | Right Cerebellum part 7b |
|  | 103 | C8.L | Left Cerebellum part 8 |
|  | 104 | C8.R | Right Cerebellum part 8 |
|  | 105 | C9.L | Left Cerebellum part 9 |
|  | 106 | C9.R | Right Cerebellum part 9 |
|  | 107 | C10.L | Left Cerebellum part 10 |
|  | 108 | C10.R | Right Cerebellum part 10 |
|  | 109 | V12 | Vermis part 1 & 2 |
|  | 110 | V3 | Vermis part 3 |
|  | 111 | V45 | Vermis part 4 & 5 |
|  | 112 | V6 | Vermis part 6 |
|  | 113 | V7 | Vermis part 7 |
|  | 114 | V8 | Vermis part 8 |
|  | 115 | V9 | Vermis part 9 |
|  | 116 | V10 | Vermis part 10 |
